# From Steklov to Neumann via homogenisation

**DOI:** 10.1007/s00205-020-01588-2

**Published:** 2020-11-20

**Authors:** Alexandre Girouard, Antoine Henrot, Jean Lagacé

**Affiliations:** 1 Département de mathématiques et de statistique, Pavillon Alexeandre-Vachon, Université Laval, Québec, QC G1V 0A6 Canada; 2 CNRS, IECL, Université de Lorraine, 54000 Nancy, France; 3 Department of Mathematics, University College London, Gower Street, London, WC1E 6BT UK

## Abstract

We study a new link between the Steklov and Neumann eigenvalues of domains in Euclidean space. This is obtained through an homogenisation limit of the Steklov problem on a periodically perforated domain, converging to a family of eigenvalue problems with dynamical boundary conditions. For this problem, the spectral parameter appears both in the interior of the domain and on its boundary. This intermediary problem interpolates between Steklov and Neumann eigenvalues of the domain. As a corollary, we recover some isoperimetric type bounds for Neumann eigenvalues from known isoperimetric bounds for Steklov eigenvalues. The interpolation also leads to the construction of planar domains with first perimeter-normalized Stekov eigenvalue that is larger than any previously known example. The proofs are based on a modification of the energy method. It requires quantitative estimates for norms of harmonic functions. An intermediate step in the proof provides a homogenisation result for a transmission problem.

## Introduction

Let $\Omega \subset {\mathbb {R}}^d$ be a bounded and connected domain with Lipschitz boundary $\partial \Omega$. Consider on $\Omega$ the Neumann eigenvalue problem

1 $$\begin{aligned} {\left\{ \begin{array}{ll} -\Delta f= \mu f&{} \text{ in } \Omega ,\\ \partial _\nu f= 0 &{} \text{ on } \partial \Omega , \end{array}\right. } \end{aligned}$$

as well as the Steklov eigenvalue problem

2 $$\begin{aligned} {\left\{ \begin{array}{ll} \Delta u=0&{} \text{ in } \Omega ,\\ \partial _\nu u=\sigma u&{} \text{ on } \partial \Omega . \end{array}\right. } \end{aligned}$$

Here $\Delta$ is the Laplacian, and $\partial _\nu$ is the outward pointing normal derivative. Both problems consist in finding the eigenvalues $\mu$ and $\sigma$ such that there exist non-trivial smooth solutions to the boundary value problems (1) and (2). For both problems, the spectra form discrete unbounded sequences

$$\begin{aligned} 0 = \mu _0 < \mu _1 \leqq \mu _2 \leqq \cdots \nearrow \infty \end{aligned}$$

and

$$\begin{aligned} 0=\sigma _0<\sigma _1\leqq \sigma _2\leqq \cdots \nearrow \infty , \end{aligned}$$

where each eigenvalue is repeated according to multiplicity. The corresponding eigenfunctions $\left\{ f_k \right\}$ and $\left\{ u_k \right\}$ have natural normalisations as orthonormal bases of $\mathrm {L}^2(\Omega )$ and $\mathrm {L}^2(\partial \Omega )$, respectively.

### From Steklov to Neumann : heuristics

Let us start by painting with a broad brush the relationships between the Neumann and Steklov eigenvalue problems; they exhibit many similar features, and it is not a surprise that they do so. Indeed, in both cases the eigenvalues are those of a differential or pseudo-differential operator, namely the Laplacian and the Dirichlet-to-Neumann map, whose kernels consist of constant functions. Moreover, in both cases, the natural isoperimetric type problem consists in maximizing $\mu _k$ and $\sigma _k$ (instead of minimizing it as is usual for the Dirichlet problem). The relation between the two boundary value problems is not solely heuristic and incidental. Indeed, it is known from the works of Arrieta–Jiménez-Casas–Rodriguez-Bernal [3] and Lamberti–Provenzano [30, 31] that one can recover the Steklov problem as a limit of weighted Neumann problems

3 $$\begin{aligned} {\left\{ \begin{array}{ll} -\Delta f= \mu \rho _\varepsilon f&{} \text{ in } \Omega ,\\ \partial _\nu f= 0 &{} \text{ on } \partial \Omega , \end{array}\right. } \end{aligned}$$

where $\rho _\varepsilon$ is a density function whose support converges to the boundary as $\varepsilon \rightarrow 0$. If we are to interpret the Neumann problem as finding the frequencies and modes of vibrations of a free boundary membrane, this means that the Steklov problem represents the frequencies and modes of a membrane whose mass is concentrated at the boundary. The reader should also refer to the work of Hassannezhad–Miclos [25, Section 4], where a similar construction is used to prove a Cheeger-type inequality for Steklov eigenvalues of a compact Riemannian manifold with boundary.

Our primary goal in this paper is to establish a link in the reverse direction, by realizing the Neumann problem as a limit of appropriate Steklov problems. This is achieved in two steps. The first one is to accumulate uniformly distributed boundary elements inside the domain $\Omega$. This is done by perforating the interior of the domain with small holes that are uniformly distributed. On these new boundary components, we consider the Steklov boundary conditions; this step is known as *the homogenisation process*. We assume that the ratio of the radii of the holes to the distance between them is at a Cioranescu–Murat type critical regime. Then, the eigenvalues and eigenfunctions of the Steklov problem converge to those of a *dynamical eigenvalue problem*

4 $$\begin{aligned} {\left\{ \begin{array}{ll} -\Delta U= A_{d} \beta \Sigma U&{} \text{ in } \Omega ,\\ \partial _\nu U=\Sigma U&{} \text{ on } \partial \Omega , \end{array}\right. } \end{aligned}$$

where $\beta \geqq 0$ is the critical regime parameter and $A_d$ is the area of the unit sphere in ${\mathbb {R}}^{d}$. Its eigenvalues form a discrete unbounded sequence:

$$\begin{aligned} \Sigma _{0,\beta }<\Sigma _{1,\beta }\leqq \Sigma _{2,\beta }\leqq \cdots \nearrow \infty ; \end{aligned}$$

once again the functions associated to the eigenvalue $\Sigma _{0,\beta } = 0$ are constant.

#### Remark 1

In [42], Joachim von Below and Gilles François studied an eigenvalue problem that is equivalent to Problem (4), which stems from a parabolic equation with dynamical boundary conditions. Indeed, they study the eigenvalue problem

5 $$\begin{aligned} {\left\{ \begin{array}{ll} - \Delta u = \lambda u &{} \text {in } \Omega ,\\ \partial _\nu u = \lambda \alpha u &{} \text {on } \partial \Omega . \end{array}\right. } \end{aligned}$$

If $\lambda$ is an eigenvalue of Problem (5), then $\Sigma =\alpha ^{-1} \lambda$ is an eigenvalue of Problem (4) with parameter $\beta = \frac{1}{\alpha A_d}$.

**Fig. 1:**
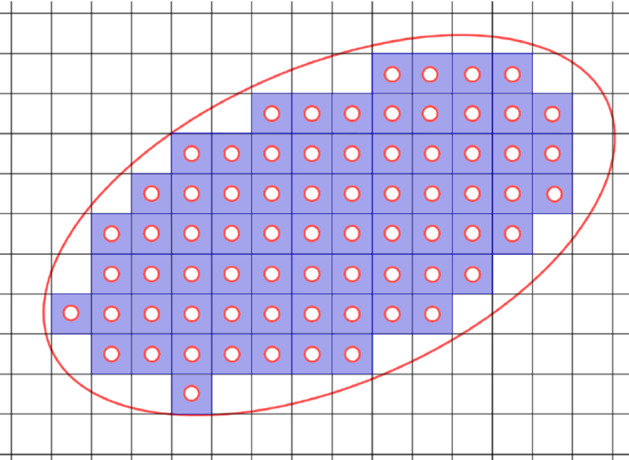
The domain $$\Omega _\varepsilon $$

The parameter $\beta$ in (4) can be interpreted as a weight on the interior of the domain, with the boundary $\partial \Omega$ having constant weight 1. In order to recover the Neumann problem, the second step will therefore be to send the parameter $\beta$ to $\infty$, putting all the weight inside the domain. Under an appropriate normalisation, eigenvalues and eigenfunctions of Problem (4) converge to those of Problem (1), completing the circle for the relation between the Steklov and the Neumann problems (Fig. 1).

### The homogenisation process

Consider a family of problems obtained by removing periodically placed balls from the domain $\Omega$. More precisely, given $0< \varepsilon < 1$, and ${\mathbf {k}}\in {\mathbb {Z}}^d$, define the cube

$$\begin{aligned} Q_{{\mathbf {k}}}^\varepsilon := \varepsilon {\mathbf {k}}+\left[ -\frac{\varepsilon }{2},\frac{\varepsilon }{2}\right] ^d\subset {\mathbb {R}}^d, \end{aligned}$$

and define the set of indices

$$\begin{aligned} I^\varepsilon := \left\{ {\mathbf {k}}\in {\mathbb {Z}}^d\,:\,Q_{{\mathbf {k}}}^\varepsilon \subset \Omega \right\} . \end{aligned}$$

Let $r_\varepsilon$ be an increasing positive function of $\varepsilon$ with $r_\varepsilon < \varepsilon /2$. For ${\mathbf {k}}\in {\mathbb {Z}}^d$, define

$$\begin{aligned} T_{{\mathbf {k}}}^\varepsilon :=B\left( \varepsilon {\mathbf {k}},r_\varepsilon \right) \subset Q_{{\mathbf {k}}}^\varepsilon \end{aligned}$$

and set

6 $$\begin{aligned} T^\varepsilon := \bigcup _{{\mathbf {k}}\in I^\varepsilon } T^\varepsilon _{\mathbf {k}}\subset \Omega . \end{aligned}$$

Consider the family of perforated domains Fig. 1.

$$\begin{aligned} \Omega ^\varepsilon =\Omega {\setminus } \overline{T^\varepsilon }. \end{aligned}$$

The Steklov eigenvalues of $\Omega ^\varepsilon$ are written as $\sigma _k^\varepsilon :=\sigma _k(\Omega ^\varepsilon ),$ and we write $\left\{ u_k^{\varepsilon } \right\}$ for a corresponding complete sequence of eigenfunctions, normalized by

$$\begin{aligned} \int _{\partial \Omega ^\varepsilon }(u_k^{\varepsilon })^2\, \mathrm {d}A=1. \end{aligned}$$

Our first main result is the following critical regime homogenisation theorem for the Steklov problem:

#### Theorem 2

Suppose that $r_\varepsilon ^{d-1} \varepsilon ^{-d} \rightarrow \beta$ for some $\beta \in [0,\infty )$, as $\varepsilon \searrow 0$. Then $\sigma _k^{\varepsilon }$ converges to the eigenvalue $\Sigma _{k,\beta }$ of (4). The functions $U_k^{\varepsilon }\in \mathrm {H}^1(\Omega )$ obtained by harmonic extension of a normalized Steklov eigenfunction $u_k^{\varepsilon }$ over the holes $T^\varepsilon \subset \Omega$ form a sequence which strongly converges in $\mathrm {H}^1(\Omega )$ to a solution $U_k$ associated with $\Sigma _{k,\beta }$ of (4).

#### Remark 3

If the eigenvalue $\Sigma _k=\Sigma _{k,\beta }$ is multiple of multiplicity *m*, *that is*

$$\begin{aligned} \Sigma _{k-1}< \Sigma _k = \cdots = \Sigma _{k+m-1} < \Sigma _{k+m}, \end{aligned}$$

then the convergence statement in the previous theorem is understood in the following sense: given a basis $U_k,\dotsc ,U_{k+m-1}$ for the eigenspace associated with $\Sigma _k$, there is a family of $m\times m$ orthogonal matrices $M(\varepsilon )$ such that

7 $$\begin{aligned} M(\varepsilon ) \begin{pmatrix} U_k^\varepsilon \\ \vdots \\ U_{k+m-1}^\varepsilon \end{pmatrix} \longrightarrow \begin{pmatrix} U_k \\ \vdots \\ U_{k+m-1} \end{pmatrix} \end{aligned}$$

as $\varepsilon \rightarrow 0$. One could also be content with the weaker statement that if the eigenvalues are multiple, the convergence statement of Theorem 2 is only true up to taking a subsequence.

#### Remark 4

Literature on homogenisation theory is often concerned with the situation where holes are proportional to their reference cell. That is, $r_\varepsilon =c\varepsilon$ for some constant $c\in (0,1/2)$. In this case one has $r_\varepsilon ^{d-1} \varepsilon ^{-d}\rightarrow \infty$. It follows from [11] that $\sigma _k^\varepsilon \rightarrow 0$. Indeed it is proved there that any bounded domain $\Omega \subset {\mathbb {R}}^d$ satisfies

8 $$\begin{aligned} \sigma _k(\Omega )\left|\partial \Omega \right|^{\frac{1}{d-1}} \leqq C_{d,k}, \end{aligned}$$

where the number $C_{d,k}>0$ depend only on the dimension *d* and index *k*. The hypothesis that $r_\varepsilon ^{d-1} \varepsilon ^{-d}\rightarrow \infty$ implies that $\left|\partial \Omega ^\varepsilon \right| \rightarrow \infty$, which forces $\sigma _k^\varepsilon \rightarrow 0$, as claimed. Note that this also corresponds to the homogenisation regime which was studied by Vanninathan in [41] for a slightly different problem, for which the Dirichlet boundary condition was imposed on $\partial \Omega$ and the Steklov condition on $\partial T^\varepsilon$.

The regime that we consider in Theorem 2 is the critical regime for the Steklov problem, where we observe a change of behaviour in the limiting problem. This is akin to the situation studied by Rauch–Taylor [38] and Cioranescu–Murat [10].

### Convergence to the Neumann problem and spectral comparison theorems

The $\beta$ parameter in Problem (4) can be interpreted as a relative weight between the interior of $\Omega$ and its boundary $\partial \Omega$ for the behaviour of that problem, see Section 2.2 for details on this interpretation. Our second main result is the following theorem, describing the specific dependence on $\beta$ in (4):

#### Theorem 5

For each $k\in {\mathbb {N}}$, the eigenvalue $\Sigma _{k,\beta }$ depends continuously on $\beta \in [0,\infty )$ and satisfies

$$\begin{aligned} \lim _{\beta \rightarrow \infty }{A_d}\beta \Sigma _{k,\beta } = \mu _k. \end{aligned}$$

The eigenfunctions $\left\{ U_{k,\beta } \right\}$ satisfy

9 $$\begin{aligned} \beta ^{1/2} U_{k,\beta } \rightarrow f_k \end{aligned}$$

strongly in $\mathrm {H}^1(\Omega )$ as $\beta \rightarrow \infty$, where $f_k$ is the *k*th non–trivial Neumann eigenfunction.

#### Remark 6

We make the second observation that this convergence *cannot* be uniform in *k*, as that would contradict [42, Theorem 4.4].

The relationships between isoperimetric type problems for the Neumann and Steklov eigenvalue problems have been investigated for the first few eigenvalues in [18, 19] from the point of view of the Robin problem. Our methods also allow us to investigate the relationship between these isoperimetric problems for every eigenvalue rank *k*.

The combination of Theorems 5 and 2 allows the transfer of known bounds for Steklov eigenvalues to bounds for Neumann eigenvalues. For instance, we can combine these two theorems with [11, Theorem 1.3], asserting that for bounded Euclidean domains with smooth boundary

10 $$\begin{aligned} \sigma _k(\Omega )|\partial \Omega |^{\frac{1}{d-1}} \leqq C(d) k^{2/d}. \end{aligned}$$

This leads to the following:

#### Corollary 7

The Neumann eigenvalues of a bounded domain $\Omega \subset {\mathbb {R}}^d$ satisfy

11 $$\begin{aligned} \mu _k(\Omega )|\Omega |^{2/d}\leqq C(d)k^{2/d}, \end{aligned}$$

where the constant *C*(*d*) is exactly that of  [11, Theorem 1.3].

#### Remark 8

The existence of a constant depending only on the dimension in inequality (11) is already known. In fact, Kröger obtained a better constant in [29]. However, it follows from Corollary 7 that any improvement to the bound (10) will transfer to bounds on Neumann eigenvalues.

One of the original motivation for this project was the study of the following quantity:

$$\begin{aligned} {\widehat{\sigma }}_k^*:=\sup \left\{ \sigma _k(\Omega )|\partial \Omega |\,:\,\Omega \subset {\mathbb {R}}^2 \text { bounded with smooth boundary}\right\} . \end{aligned}$$

In dimension $d=2$, we are able to get a stronger version of Corollary 7 in the sense that we obtain a direct link between ${\widehat{\sigma }}_k^*$ and

$$\begin{aligned} {\widehat{\mu }}_k^*:=\sup \left\{ \mu _k(\Omega )|\Omega |\,:\,\Omega \subset {\mathbb {R}}^2 \text { bounded with smooth boundary}\right\} . \end{aligned}$$

In that case, we obtain:

#### Theorem 9

For $d = 2$ and every $k \in {\mathbb {N}}$,

12 $$\begin{aligned} {\widehat{\mu }}_k^* \leqq {\widehat{\sigma }}_k^*. \end{aligned}$$

#### Remark 10

From [28], we have that

$$\begin{aligned} \sigma _1(\Omega ) \left|\partial \Omega \right| \leqq 8 \pi . \end{aligned}$$

It follows from Theorem 9 that

$$\begin{aligned} \mu _1(\Omega ) \left|\Omega \right| \leqq 8 \pi , \end{aligned}$$

Of course, this bound is already known. Indeed the optimal upper bound is given by the famous Szegő–Weinberger theorem,

$$\begin{aligned} \mu _1({\mathbb {D}})\pi \approx 3.39\pi , \end{aligned}$$

however, it exemplifies the general principle that any bound for $\sigma _k$ normalised by perimeter transfers to bounds for $\mu _k$ normalised by area.

The previous discussion also yields the following corollary:

#### Corollary 11

$$\begin{aligned} {\widehat{\sigma }}_1^*\geqq \mu _1({\mathbb {D}})\times \pi \approx 3.39\pi . \end{aligned}$$

Indeed, by the Szegő–Weinberger inequality, we have that

13 $$\begin{aligned} {\widehat{\sigma }}^*_1 \geqq \pi \mu _1({\mathbb {D}}) \approx 3.39 \pi . \end{aligned}$$

Furthermore, this number will be approached as close as desired in homogenisation sequence of pierced unit disks with large enough parameter $\beta$. Of course, it is known from [17] that if one is allowed to optimise amongst surfaces rather than Euclidean domains, then there is a sequence of surfaces such that $\sigma _1(\Omega _n)\left|\partial \Omega _n \right| \rightarrow 4\pi$. This leads to the following natural conjecture:^1^

#### Conjecture

$$\begin{aligned} {\widehat{\sigma }}_1^* = \mu _1({\mathbb {D}})\times \pi \approx 3.39\pi . \end{aligned}$$

Note that the previous best known lower bound for ${\widehat{\sigma }}_1^*$ was attained on some concentric annulus, whose first normalised Steklov eigenvalue is approximately $2.17 \pi$, see [22]. We also observe that a similar analysis yields that for any $\Omega \subset {\mathbb {R}}^2$ bounded with smooth boundary,

$$\begin{aligned} {\widehat{\sigma }}_k^* \geqq \mu _k(\Omega ) \left|\Omega \right|. \end{aligned}$$

In particular, it follows from Weyl’s law for Neumann eigenvalues that there exists a sequence $a_k \sim 4\pi k$ such that

$$\begin{aligned} {\widehat{\sigma }}_k^* \geqq a_k. \end{aligned}$$

### Discussion

Homogenisation theory is a young branch of mathematics which started around the 1960’s. Its general goal is to describe macroscopic properties of materials through their microscopic structure. To the best of our knowledge, the first papers to study periodically perforated domains from a rigourous mathematical point of view are those of Marchenko and Khruslov from the early 1960’s (for example [33]) leading to their influential book [34] in 1974. The topic became widely known in the West with the work of Rauch and Taylor on the *crushed ice problem* [38] in 1975 and then with the publication in 1982 of [10] by Cioranescu and Murat. Many of these early results were concerned with the Poisson problem $\Delta u_\varepsilon =f$ under Dirichlet boundary conditions $u_\varepsilon =0$ on $\partial \Omega _\varepsilon$. The limiting behaviour of the solution $u_\varepsilon$ depends on the rate at which $r_\varepsilon \searrow 0$. Three regimes are considered. If the size of the holes $r_\varepsilon$ tends to zero very fast, then in the limit the solutions tend to those of the Poisson problem on the original domain $\Omega$, while if the size of the holes are big enough the solutions tend to zero. The main interest comes from the critical regime, in which case the solutions tend to solutions of a new elliptic problem.

In this paper we are concerned with the much less studied Steklov spectral problem (2). From the point of view of homogenisation theory, this problem is atypical in the sense that the function spaces which occur for different values of the parameter $\varepsilon >0$ are not naturally related. This means, in particular, that this problem does not yield to any of the usual general frameworks used in homogenisation theory (such as [26, Chapter 11]). Nevertheless, several authors have considered homogenisation for this problem, using ad hoc methods depending on the specifc situation considered. The behaviour of Steklov eigenvalues under singular perturbations such as the perforation of a single hole has also been studied in [24, 36].

Our main inspiration for this work is the paper [41]. Several papers have also considered homogenisation in the situation where the Dirichlet condition is imposed on the outer boundary while the Steklov condition is considered only on the boundary of the holes [8, 14]. In these papers the holes are proportional to the size of the reference cell. The novelty of our homogenisation result in the case of the Steklov problem is that consider holes that are shrinking much faster than that, in a critical regime where the limiting problem is fundamentally different. They are in fact shrinking at the precise rate which makes their total surface area (or perimeter in dimension 2) is asymptotically comparable with the volume of the domain. This is similar to the work of Rauch–Taylor [38] for the Neumann problem, Cioranescu–Murat [10] for the Dirichlet problem and Kaizu for the Robin problem [27].

The *energy method* of Tartar (see [1, Section 1.3] for an exposition) has been used extensively in the study of homogenisation problems at critical regimes, see [10, 27] and more recently [7], where they obtain norm-resolvant convergence for the Dirichlet, Neumann and Robin problem. That method uses an auxiliary function, satisfying some energy-minimising PDE in the fundamental cells, in order to derive convergence of the problem in the weak formulation. The method, in its Robin or Neumann form, is boundary-condition agnostic and as such is ill-suited for the Steklov problem, where the normalisation is with respect to $\mathrm {L}^2(\partial \Omega ^\varepsilon )$. Indeed, while the technique could be used to obtain some form of convergence, it will not be able to transfer boundary estimates to $\mathrm {L}^2(\Omega )$, and ensure that the limit solution doesn’t degenerate to the trivial one.

Nevertheless, one can interpret our technique as a variation on the energy method, adapted for problems defined on the boundary. We are also using an auxiliary PDE in order to derive convergence, but it does not stem from compensated compactness. The main difference, however, is that we can deduce interior estimates from those on the boundary of the periodic holes from our auxiliary problem, see Lemma 26.

We note that in this paper we have chosen to consider only spherical holes. In fact, it should be possible to consider more general convex holes obtained as scaled copies $r_\varepsilon \omega$ of a fixed convex set $\omega$, as it is done in classical homogenization literature. Here, convexity of the holes would be required for $\mathrm {L}^\infty$ estimates of the Steklov eigenfunctions; see Lemma 16.

Since we are motivated by spectral questions, namely to explore a new link between the Steklov problem and the Neumann problem, we decided to avoid the technical difficulties which would occur by considering more general inclusions. This allows us to get the simpler dynamical eigenvalue problem (3) and emphasise clearly the link with the the classical Neumann eigenvalue problem by letting the parameter $\beta \rightarrow +\infty$. Nevertheless, many of our proofs carry through with the spherical holes being replaced with convex holes, with the exception that the convergence in Theorem 2 is now weak in $\mathrm {H}^1(\Omega )$ and $A_d$ is replaced with the boundary measure of the unscaled hole. The convexity assumption is used to obtain bounds on the $\mathrm {L}^\infty$ norm of the eigenfunctions; they are more sensitive to the shape of the boundary than, say, Neumann eigenfunctions. Convergence of the energy, and therefore strong convergence, would require a finer analysis of correctors which we decided to avoid for the aforementioned reasons.

### Structure of the proof and plan of the paper

In Section 2 we formally describe properties of the various eigenvalue problems that we study as well as the functions spaces over which they are defined. While they are well-known, the notation used for all of them often collides. In that section, we fix notation once and for all for the remainder of the paper for definedness and ease of references.

In Section 3 we study one of the main technical tools in this paper, properties of harmonic extensions of functions on annuli to the interior disk. Results are separated into two categories: those that rely on the fact that the functions satisfy a Robin-type boundary condition, and more general results that do not rely on such a thing. Most of our results will be obtained by considering the Fourier expansion of a function

$$\begin{aligned} u(\rho ,{\varvec{\theta }}) = \sum _{\ell ,m} a_{\ell }^m(\rho ) Y_\ell ^m({\varvec{\theta }}) \end{aligned}$$

in spherical harmonics, and obtaining our inequalities term by term for every $a_\ell ^m$.

Section 4 is the *pièce de résistance* of this paper. It is where we show Theorem 2 and the proof proceeds in many steps. We first prove that the family of harmonic extensions $U^\varepsilon :=U_k^\varepsilon$ is bounded in the Sobolev space $H^1(\Omega )$ hence there exists a subsequence $\varepsilon _n\searrow 0$ such that $U^{\varepsilon _n}$ weakly converges to a function $U\in H^1(\Omega )$. This allows us to consider properties of the weak limit *U*, and of an associated limit $\Sigma$ to the eigenvalue sequence $\sigma _k^{\varepsilon _n}$.

It is then not so hard to show that, using the weak formulations of Problems (2) and (4) that the limit of the homogenised Steklov problem contains terms corresponding to the limit dynamical eigenvalue problem, plus some spurious terms that must be shown to converge to zero. This is done by studying two representations of the weak formulation using Green’s identity either towards the inside of the holes or the inside of the domains $\Omega ^\varepsilon$. The first one is used to show that the functionals that arise in the study of the homogenisation problem are uniformly bounded, hence we can use smooth test functions. In the second representation, we are therefore allowed to use smooth test functions, which allows us to recover convergence to zero of the spurious terms. A key step in this argument is to understand the limit behaviour of an auxiliary homogenisation problem for the transmission eigenvalue problem (see Proposition 20).

Once we have established convergence to a solution of Problem 4, we end up showing that the limit eigenpair $(\Sigma ,U)$ does not degenerate to the trivial function. Using variational characterisation of eigenvalues and eigenfunctions, we can also show that we get complete spectral convergence, and that subsequences are not needed.

Finally, in Section 5 we show convergence to the Neumann problem as $\beta \rightarrow \infty$. The method is similar to the one used at the end of the previous section, but many of the inequalities are more subtle. We also show the comparison theorems between Steklov and Neumann eigenvalues in this section.

In this paper, we use *c* and *C* to mean constants whose precise value is not important to our argument, and whose exact value may change from line to line. We use the notations $f = O_{}\left( g \right)$ and $f \ll g$ interchangeably to mean that there exists a constant *C* such that $\left|f(x) \right| \leqq Cg(x)$.

## Notation and function spaces

Four different eigenvalue problems will be used. The goal of this section is to introduce them and fix the relevant notation. Throughout the paper, we use real valued functions.

### The Steklov problem on $\Omega$ and $\Omega ^\varepsilon$

Given a bounded domain $\Omega$ whose boundary $\partial \Omega$ is smooth, the Dirichlet-to-Neumann operator (DtN map) $\Lambda$ acts on $C^\infty (\partial \Omega )$ as

14 $$\begin{aligned} \Lambda f = \partial _\nu {\widehat{f}}, \end{aligned}$$

where ${\widehat{f}}$ is the harmonic extension of *f* to the interior of $\Omega$. The DtN map is an elliptic, positive, self-adjoint pseudodifferential operator of order 1. Because $\partial \Omega$ is compact, it follows from standard theory of such operators, see *e.g* [39], that the eigenvalues form a non–negative unbounded sequence $\left\{ \sigma _k : k \in {\mathbb {N}}_0 \right\}$ and that there exists an orthonormal basis of $(f_k)$ of $\mathrm {L}^2(\partial \Omega )$ such that $\Lambda f_k=\sigma _kf_k$. The harmonic extensions $u_k={\widehat{f}}_k$ satisfy the Steklov problem

15 $$\begin{aligned} {\left\{ \begin{array}{ll} - \Delta u_k = 0 &{} \text {in } \Omega ,\\ \partial _\nu u_k = \sigma _k u_k &{}\text {on } \partial \Omega . \end{array}\right. } \end{aligned}$$

In general, we use the same symbol $u_k$ for the function on $\Omega$ and for its trace on the boundary $\partial \Omega$. The eigenvalue sequence for $\Omega ^\varepsilon$ is denoted $\left\{ \sigma _k^\varepsilon : k \in {\mathbb {N}}_0) \right\}$, with corresponding eigenfunctions $u_k^\varepsilon$. The eigenfunctions $u_k$ and $u_k^\varepsilon$ form an orthonormal basis with respect to the inner products

16 $$\begin{aligned} (f,g)_\partial := \int _{\partial \Omega } f g \, \mathrm {d}A, \end{aligned}$$

and

17 $$\begin{aligned} (f,g)_{\partial ^\varepsilon } := \int _{\partial \Omega ^\varepsilon } f g \, \mathrm {d}A. \end{aligned}$$

The *k*-th nonzero eigenvalue $\sigma _k$ is characterised by

18 $$\begin{aligned} \sigma _k = \inf \left\{ \frac{\int _\Omega \left|\nabla u \right|^2 \, \mathrm {d}{\mathbf {x}}}{\int _{\partial \Omega }u^2 \, \mathrm {d}A}:u \in \mathrm {H}^1(\Omega ) \text { and } (u,u_j)_{\partial }=0 \text{ for } 0 \leqq j < k \right\} . \end{aligned}$$

The eigenvalues $\sigma _k^\varepsilon$ have the same characterisation, integrating over $\Omega ^\varepsilon$ and $\partial \Omega ^\varepsilon$ respectively instead, and with the orthogonality being with respect to $(\cdot ,\cdot )_{\partial ^\varepsilon }$.

### Dynamical eigenvalue problem

For $\beta \in (0,\infty )$, consider the eigenvalue problem

19 $$\begin{aligned} {\left\{ \begin{array}{ll}-\Delta U = A_d \beta \Sigma U &{} \text {in } \Omega ,\\ \partial _\nu U = \Sigma U &{} \text {on } \partial \Omega , \end{array}\right. } \end{aligned}$$

where $A_d$ is the area of the unit sphere in ${\mathbb {R}}^d$. Problem (19) was introduced with a slightly different normalization in [42], where it is called a *dynamical eigenvalue problem*. The eigenvalues and eigenfunctions are those of the operator

20 $$\begin{aligned} P := \begin{pmatrix} -(A_d\beta )^{-1} \Delta &{} 0 \\ 0 &{} \partial _\nu \end{pmatrix}. \end{aligned}$$

This unbounded operator is defined on an appropriate domain in the space $\mathrm {L}^2_{A_d \beta }(\Omega ) \times \mathrm {L}^2 (\partial \Omega )$ which consists simply of $\mathrm {L}^2(\Omega ) \times \mathrm {L}^2 (\partial \Omega )$ equipped with the inner product defined by

21 $$\begin{aligned} (f,g)_\beta := A_d \beta \int _\Omega f g \, \mathrm {d}{\mathbf {x}}+ \int _{\partial \Omega } f g \, \mathrm {d}A. \end{aligned}$$

The dynamical eigenvalue problem (19) has a discrete sequence of eigenvalues

22 $$\begin{aligned} 0 = \Sigma _{0,\beta } < \Sigma _{1,\beta } \leqq \Sigma _{2,\beta } \leqq \cdots \nearrow \infty . \end{aligned}$$

Let $X\subset \mathrm {L}^2_{A_d \beta }(\Omega ) \times \mathrm {L}^2 (\partial \Omega )$ be the subspace defined by

23 $$\begin{aligned} X:= \left\{ U = (u,\tau u) : u \in \mathrm {H}^1(\Omega ) \right\} , \end{aligned}$$

where $\tau :\mathrm {H}^1(\Omega ) \rightarrow \mathrm {L}^{2}(\partial \Omega )$ is the trace operator. The eigenfunctions $U_{k,\beta }$ associated to $\Sigma _{k,\beta }$ form a basis of *X*, but not of $\mathrm {L}^2_{A_d\beta }(\Omega ) \times \mathrm {L}^2(\partial \Omega )$. The eigenvalues $\Sigma _{k,\beta }$ are characterised by

24 $$\begin{aligned} \Sigma _{k,\beta } = \inf \left\{ \frac{\int _\Omega \left|\nabla U \right|^2 \, \mathrm {d}{\mathbf {x}}}{A_d \beta \int _\Omega U^2 \, \mathrm {d}{\mathbf {x}}+ \int _{\partial \Omega } U^2 \, \mathrm {d}A}:U \in \mathrm {H}^1(\Omega ) \text { and } (U,U_{j,\beta })_{\partial }=0 \text{ for } 0 \leqq j < k \right\} .\nonumber \\ \end{aligned}$$

### The Neumann eigenvalue problem

We will also make use of the classical Neumann eigenvalue problem

25 $$\begin{aligned} {\left\{ \begin{array}{ll}-\Delta f = \mu f &{} \text {in } \Omega ,\\ \partial _\nu f = 0 &{} \text {on } \partial \Omega . \end{array}\right. } \end{aligned}$$

The Neumann eigenvalues form an increasing sequence

26 $$\begin{aligned} 0 = \mu _0 < \mu _1 \leqq \mu _2 \leqq \cdots \infty , \end{aligned}$$

with associated eigenfunctions $f_k$, orthonormal with respect to the $\mathrm {L}^2(\Omega )$ inner product

27 $$\begin{aligned} (f,g) := \int _\Omega f g \, \mathrm {d}{\mathbf {x}}. \end{aligned}$$

The eigenvalues are characterised by

28 $$\begin{aligned} \mu _k = \inf \left\{ \frac{\int _\Omega \left|\nabla f \right|^2\, \mathrm {d}{\mathbf {x}}}{\int _{\Omega } f^2 \, \mathrm {d}{\mathbf {x}}}: f \in \mathrm {H}^1(\Omega ) \text { and } (f,f_j)=0 \text{ for } j=0,1,\cdots ,k-1 \right\} .\nonumber \\ \end{aligned}$$

## Comparison theorems

In this section, we derive comparison inequalities that will be used repeatedly. For $0< r < R$, set

$$\begin{aligned} A_{r,R} = B(0,R) {\setminus }\overline{B(0,r)}. \end{aligned}$$

For any set *X* where it is defined, the Dirichlet energy of a function $f:X\rightarrow {\mathbb {R}}$ is

$$\begin{aligned} {\mathcal {D}}(f) :=\int _{X}|\nabla f|^2\,\, \mathrm {d}{\mathbf {x}}. \end{aligned}$$

The Dirichlet energy of *f* on a subset $Y\subset X$ is written ${\mathcal {D}}(f;Y)$. Note that since every problem under consideration is self-adjoint no generality is lost by studying real-valued functions.

Lemmas 12 and 13 are proved by using a Fourier series decomposition for functions in an annulus. We note that even though the proofs rely on estimates on the radial part of these functions, we do not claim that the inequalities proved therein are realised by radial optimisers. The standard Schwarz symmetrisation arguments do not apply here because the domain of the functions are annuli rather than balls. Furthermore, in several similar situations some breaking of symmetry occurs: radially symmetric minimisation problems have nonradial minimisers. We refer, for instance, to the work of Esteban [15] and Lopes [32, Section III] for such examples in settings close to ours.

### Comparison theorems for functions satisfying a Steklov boundary condition

Our first comparison result concerns the Dirichlet energy of the harmonic extension of functions satisfying a Steklov boundary condition on the inner boundary of an annulus. For a similar but weaker result, ultimately sufficient to obtain weak convergence in Theorem 2, we refer the reader to [38, Example 1, p. 40] where an argument relying purely on scaling is given.

#### Lemma 12

Fix a positive real number $\sigma >0$. For any $0< r < R \leqq 1$, let $u\in C^\infty (\overline{A_{r,R}})$ be such that

29 $$\begin{aligned} {\left\{ \begin{array}{ll} \Delta u=0&{} \text{ in } A_{r,R},\\ \partial _\nu u=\sigma u&{} \text{ on } \partial B(0,r). \end{array}\right. } \end{aligned}$$

Consider the function $h:B(0,r)\rightarrow {\mathbb {R}}$ defined by

$$\begin{aligned}{\left\{ \begin{array}{ll} h=u&{} \text{ on } \partial B(0,r),\\ \Delta h=0&{} \text{ in } B(0,r). \end{array}\right. } \end{aligned}$$

Then as the ratio *r*/*R* goes to 0,

30 $$\begin{aligned} {\mathcal {D}}(h) \leqq 5 {\mathcal {D}}(u) \left( \frac{r}{R} \right) ^d \left( 1 + O_{}\left( \left( \frac{r}{R} \right) ^d \right) \right) . \end{aligned}$$

#### Proof

For every $\ell \geqq 0$, we denote by $N_\ell$ the dimension of the space $H_\ell$ of spherical harmonics of order $\ell$ and denote by $Y_\ell ^m({\varvec{\theta }})$, $1 \leqq m \leqq N_\ell$ the standard orthonormal basis of spherical harmonics on the unit sphere. On $A_{r,R}$, the function *u* admits a Fourier decomposition in spherical harmonics

31 $$\begin{aligned} u(\rho ,{\varvec{\theta }}) = \sum _{\begin{array}{c} \ell \geqq 0 \\ 1 \leqq m \leqq N_\ell \end{array}} a_\ell ^m(\rho ) Y_\ell ^m({\varvec{\theta }}). \end{aligned}$$

We start by studying the form of the coefficients $a_\ell ^m$.

**Case** $d>2$. The harmonicity condition on *u* implies that the radial parts $a_\ell ^m(\rho )$ are given by

32 $$\begin{aligned} a_\ell ^m(\rho ) = c_\ell ^m \rho ^{\ell } + c_{-\ell }^m \rho ^{-\ell + 2 - d}. \end{aligned}$$

By convention the coefficients $c_0^1$ and $c_{-0}^1$ are assumed to be different, the minus sign referring as for the other coefficients to the solution blowing up at the origin. The Steklov condition for *u* on $\partial B(0,r)$ along with the orthogonality of the spherical harmonics $Y_\ell ^m$ imply

33 $$\begin{aligned} - \partial _\rho a_\ell ^m(r) = \sigma a_\ell ^m(r), \end{aligned}$$

which yields the relations

34 $$\begin{aligned} c_{-\ell }^m = \overbrace{\left( \frac{\ell + r \sigma }{\ell - 2 + d - r \sigma }\right) }^{M:=}r^{2\ell +d-2}c_\ell ^m. \end{aligned}$$

In turns this yields the following explicit expression for the radial functions:

35 $$\begin{aligned} a_\ell ^m(\rho ) = c_\ell ^m \rho ^{\ell }\left( 1+ M \left( \frac{r}{\rho }\right) ^{2\ell +d-2}\right) . \end{aligned}$$

For $r \leqq \frac{d - 2}{2\sigma }$, it follows that

36 $$\begin{aligned} \frac{\ell }{\ell - 2 + d}\leqq M \leqq 1. \end{aligned}$$

**Case** $d=2$. Equations (32) holds except at $\ell = 0$, in which case,

$$\begin{aligned} a_0^1(\rho ) = c_0^1 + c_{-0}^1 \log \rho . \end{aligned}$$

In that case, (35) becomes

$$\begin{aligned} a_0^1&= c_0^1 \left( 1 + \frac{r\sigma }{1 + r \log r} \log (1/\rho ) \right) \\&= c_0^1 \left( 1 + M \log (1/\rho )\right) . \end{aligned}$$

Observe for the sequel that when $d = 2$ and $\ell = 0$, then $M = O_{}\left( r\sigma \right)$, and if $\ell > 0$, then

37 $$\begin{aligned} M = 1 + O_{}\left( r \sigma \right) . \end{aligned}$$

Because inequality (30) is invariant under scaling, it is sufficient to prove the case $R= 1$ and to let $r \rightarrow 0$. The harmonic extension of *u* to *B*(0, *r*) is given by

38 $$\begin{aligned} h(\rho ,{\varvec{\theta }}) = \sum _{\begin{array}{c} \ell \geqq 0 \\ 1 \leqq m \leqq N_\ell \end{array}} a_\ell ^m(r) \frac{\rho ^\ell }{r^\ell } Y_\ell ^m({\varvec{\theta }}). \end{aligned}$$

The Dirichlet energy of *h* is

39 $$\begin{aligned} {\mathcal {D}}(h) = \sum _{\begin{array}{c} \ell \geqq 1 \\ 1 \leqq m \leqq N_\ell \end{array}} \ell a_\ell ^m(r)^2 r^{d-2}. \end{aligned}$$

On the other hand, the Dirichlet energy of *u* is given, from Green’s identity, and the Steklov condition on $\partial B(0,r)$

40 $$\begin{aligned} {\mathcal {D}}(u) = \sum _{\ell ,m} \sigma a_\ell ^m(r)^2r^{d-1} + \sum _{\ell ,m} a_\ell ^m(1)\partial _\rho a_\ell ^m(1). \end{aligned}$$

Our goal is now to find a bound on (39) in terms of (40). It is sufficient to show that for each $\ell \geqq 1$,

41 $$\begin{aligned} \ell a_\ell ^m(r)^2 \leqq 4 r^2 a_\ell ^m(1) \partial _\rho a_\ell ^m(1)\left( 1 + O_{}\left( r^d \right) \right) . \end{aligned}$$

Suppose without loss of generality that $c_\ell ^m = 1$. The substitution of (34) into (32) imply that

42 $$\begin{aligned} a_\ell ^m(r)^2 = r^{2 \ell } \left( 1 + M \right) ^2 \leqq 5r^2, \end{aligned}$$

and that

43 $$\begin{aligned} a_\ell ^m(1)\partial _\rho a_\ell ^m(1)&= \ell \left( 1 + \frac{(2-d) M}{\ell } r^{2\ell + d - 2} + \frac{( 2 - d - \ell ) M^2}{\ell } r^{2(2 \ell + d-2)}\right) \nonumber \\&= \ell + O_{}\left( r^d \right) . \end{aligned}$$

Hence, dividing (42) by (43), and using the bounds on *M* from (36) and (37) we have that for $\ell \geqq 1$,

44 $$\begin{aligned} \ell a_\ell ^m(r)^2 \leqq 5 a_\ell ^m(1) \partial _\rho a_\ell ^m(1)r^2 \left( 1 + O_{}\left( r^d \right) \right) . \end{aligned}$$

This proves our claim. $\square$

### General $\mathrm {H}^1$ comparison theorems on annuli and balls

The next two lemmas do not depend on any specific boundary condition. We remark that all the lemmas in this section carry for convex, rather than spherical, inclusions. This is the case since all quantities at hand are bounded in terms of the Lipschitz constant of a diffeomorphism, and none of the estimates rely on solving a specific differential equation.

The first Lemma gives bounds for Sobolev constants of annuli.

#### Lemma 13

For $$0< r< R < 1$$, define

45 $$\begin{aligned} \gamma (r,R) := \inf \left\{ \frac{\int _{A_{r,R}} \left|\nabla u \right|^2 + u^2\,\, \mathrm {d}{\mathbf {x}}}{\int _{\partial B(0,r)} u^2\,dA}:u \in \mathrm {H}^1(A_{r,R}), u\big |_{\partial B(0,r)} \not \equiv 0 \right\} . \end{aligned}$$

Suppose that $R \geqq c r^{\frac{d-1}{d}} \geqq 2r$ for some $c > 0$. Then, there is a constant *C* depending only on the dimension and on *c* such that

46 $$\begin{aligned} \gamma (r,R) \geqq C \min \left\{ R^d r^{1-d},r^{\frac{1}{d} - 1} \right\} . \end{aligned}$$

#### Proof

As earlier, write a function $u \in \mathrm {H}^1(A_{r,R})$ as

47 $$\begin{aligned} u(\rho ,{\varvec{\theta }}) = \sum _{\ell ,m} a_\ell ^m(\rho ) Y_\ell ^m({\varvec{\theta }}). \end{aligned}$$

Using the notation $u_{{\varvec{\theta }}}$ for the tangential gradient, the Dirichlet energy of *u* is expressed as

48 $$\begin{aligned} \begin{aligned} {\mathcal {D}}(u)&= \int _r^R \int _{{\mathbb {S}}^{d-1}} \left( u_\rho ^2 + \rho ^{-2} u_{{\varvec{\theta }}}^2 \right) \rho ^{d-1} \, \mathrm {d}{\varvec{\theta }}\, \mathrm {d}\rho \\&\geqq \int _r^R \int _{{\mathbb {S}}^{d-1}} \left( u_\rho ^2\right) \rho ^{d-1} \, \mathrm {d}{\varvec{\theta }}\, \mathrm {d}\rho \\&= \sum _{\ell ,m} \int _r^R \left( a_\ell ^m(\rho )'\right) ^2 \rho ^{d-1} \, \mathrm {d}\rho . \end{aligned} \end{aligned}$$

On the other hand, the denominator in (45) is given by

49 $$\begin{aligned} \int _{\partial B(0,r)} u^2 \, \mathrm {d}A = r^{d-1} \sum _{\ell ,m} a_\ell ^m(r)^2. \end{aligned}$$

Combining these last two expressions in (45) and defining the density $w(\rho ) = \left( \frac{\rho }{r} \right) ^{d-1}$, we see it is enough to prove that

50 $$\begin{aligned} \sum _{\ell ,m} \int _r^R \left( \left( \partial _\rho a_\ell ^m(\rho ) \right) ^2 + a_\ell ^m(\rho )^2\right) w(\rho ) \, \mathrm {d}\rho \geqq C \min \left\{ R^d r^{1-d},r^{\frac{1}{d} - 1} \right\} \sum _{\ell ,m} a_\ell ^m(r)^2.\nonumber \\ \end{aligned}$$

Indeed, working term by term, we prove that any smooth function $f : [r,R] \rightarrow {\mathbb {R}}$ satisfies

51 $$\begin{aligned} \int _r^R \left( f'(\rho )^2 + f(\rho )^2\right) w(\rho ) \, \mathrm {d}\rho \geqq C \min \left\{ R^d r^{1-d},r^{\frac{1}{d} - 1} \right\} f(r)^2. \end{aligned}$$

To this end, assume without loss of generality that $f(r) = 1$. Following a strategy that was used in [12] and in [9], consider the two following situations.

Let $t\in (r,R)$, to be fixed later.

**Case a.** Suppose first that for all $\rho \in (t,R)$, $\left|f(\rho ) \right| \geqq 1/2$. It follows from monotonicity and explicit integration that

52 $$\begin{aligned} \int _r^R \left|f(\rho ) \right|^2 w(\rho ) \, \mathrm {d}\rho \geqq \frac{r^{1-d}}{4d}\left( R^d - t^d \right) . \end{aligned}$$

**Case b.** Suppose there exists $\rho _0 \in (t,R)$ such that $f(\rho _0) < 1/2$. Splitting the integral, using the fact that $w(\rho ) \geqq 1$ and is increasing for all $\rho$ together with the Cauchy–Schwarz inequality leads to

53 $$\begin{aligned} \begin{aligned} \int _r^R f'(\rho )^2 w(\rho ) \, \mathrm {d}\rho&\geqq \int _r^t f'(\rho )^2 + \int _t^{\rho _0} f'(\rho )^2w(\rho ) \, \mathrm {d}\rho \\&\geqq \frac{1}{t - r}\left( \int _r^t f'(\rho ) \, \mathrm {d}\rho \right) ^2 + \left( \frac{t}{r} \right) ^{d-1}\frac{1}{\rho _0 - t}\left( \int _t^{\rho _0} f'(\rho ) \, \mathrm {d}\rho \right) ^2. \end{aligned} \end{aligned}$$

By hypothesis, $R<1$, so that $\frac{1}{\rho _0-t}>1$. This leads to

54 $$\begin{aligned} \begin{aligned} \int _r^R f'(\rho )^2 w(\rho ) \, \mathrm {d}\rho \geqq \frac{1}{2} \min \left\{ \frac{1}{t - r},\left( \frac{t}{r} \right) ^{d-1} \right\} \left( \int _r^{\rho _0}f'(\rho ) \, \mathrm {d}\rho \right) ^2. \end{aligned} \end{aligned}$$

Choosing $t = \min \left\{ r^{\frac{d-1}{d}},R/2 \right\}$ guarantees that $\min \left\{ \frac{1}{t - r},\left( \frac{t}{r} \right) ^{d-1} \right\} =\left( \frac{t}{r} \right) ^{d-1}$, so that

55 $$\begin{aligned} \begin{aligned} \int _r^R \left|f'(\rho ) \right|^2 w(\rho ) \, \mathrm {d}\rho \geqq \frac{1}{2}\left( \frac{t}{r} \right) ^{d-1}\left( \int _r^{\rho _0}f'(\rho ) \, \mathrm {d}\rho \right) ^2. \end{aligned} \end{aligned}$$

It follows from the definition of *t* that

$$\begin{aligned} \left( \frac{t}{r}\right) ^{d-1}\geqq \overbrace{\min \left\{ 1,\left( \frac{c}{2}\right) ^{d-1}\right\} }^{A:=}r^{\frac{1-d}{d}}. \end{aligned}$$

We can bound asymptotically the last integral in (53) as

56 $$\begin{aligned} \begin{aligned} \int _r^R f'(\rho )^2 w(\rho ) \, \mathrm {d}\rho&\geqq Cr^{\frac{1-d}{d}} \left( \int _r^{\rho _0}f'(\rho )\, \mathrm {d}\rho \right) ^2\\&\geqq \frac{A}{4}r^{\frac{1-d}{d}}. \end{aligned} \end{aligned}$$

This also ensures that $R^d - t^d \geqq (1-2^{-d})R^d$. Since both situations are exclusive, inequality (50) holds, finishing the proof. $\square$

The next lemma compares $\mathrm {L}^2$ norms on *B*(0, *r*) with $\mathrm {H}^1$ norms on *B*(0, *R*). Here, for any $\Omega ' \subset \Omega$, the norm on $\mathrm {H}^1(\Omega ')$ is given by

57 $$\begin{aligned} \left\| u \right\| _{\mathrm {H}^1(\Omega ')}^2 = \left\| u \right\| _{\mathrm {L}^2(\Omega ')}^2 + \left\| \nabla u \right\| ^2_{\mathrm {L}^2(\Omega ')^2}. \end{aligned}$$

#### Lemma 14

For $0< r < R\leqq 1$, if $R \geqq c r^{\frac{d-1}{d}}$ for some $c>0$, there is a constant *C* depending only on *c* and on the dimension such that for all $u \in \mathrm {H}^1(B(0,R))$,

58 $$\begin{aligned} \left\| u \right\| _{\mathrm {L}^2(B(0,r))} \leqq C r^{1/2} \left\| u \right\| _{\mathrm {H}^1(B(0,R))}. \end{aligned}$$

#### Proof

Let $$u\in H^1(B(0,R))$$. Given $$r\in (0,R)$$,

$$\begin{aligned} \int _{B(0,r)}u^2\,\, \mathrm {d}{\mathbf {x}}=\int _{0}^r\rho ^{d-1}\int _{S^{d-1}}u^2\,\, \mathrm {d}\theta \,\, \mathrm {d}\rho =\int _{0}^r\Vert u\Vert _{L^2(\partial B(0,\rho ))}^2\,\, \mathrm {d}\rho \end{aligned}$$

It follows from the definition of $\gamma$ in Lemma 13 that

$$\begin{aligned} \Vert u\Vert _{\partial B(0,\rho )}^2\leqq \frac{1}{\gamma (\rho ,R)}\Vert u\Vert _{H^1(A_{\rho ,R})}^2 \leqq \frac{1}{\gamma (\rho ,R)}\Vert u\Vert _{H^1(B(0,R))}^2. \end{aligned}$$

Substitution in the above leads to

$$\begin{aligned} \int _{B(0,r)}u^2\,\, \mathrm {d}{\mathbf {x}}\leqq \Vert u\Vert _{H^1(B(0,R))}^2\int _{0}^r\frac{1}{\gamma (\rho ,R)} \, \mathrm {d}\rho . \end{aligned}$$

It follows from Lemma 13 that

$$\begin{aligned} \int _{0}^r\frac{1}{\gamma (\rho ,R)}\,\, \mathrm {d}\rho&\leqq \frac{1}{C}\int _{0}^r\left( \frac{\rho ^{d-1}}{R^d}+\rho ^{1-\frac{1}{d}}\right) \, \mathrm {d}\rho \\&=\frac{1}{C}\left( \frac{r^d}{dR^d}+\frac{r^{2-\frac{1}{d}}}{2-\frac{1}{d}}\right) \\&\leqq \frac{1}{C}\left( \frac{r}{cd}+\frac{r^{2-\frac{1}{d}}}{2-\frac{1}{d}}\right) \qquad \longleftarrow \text { since }R^d\geqq cr^{d-1}\\&\leqq {\widetilde{C}}r. \end{aligned}$$

$$\square $$

Finally, we require the following lemma about the behaviour of the boundary trace operator as a domain gets shrunk:

#### Lemma 15

Let $\Omega \subset {\mathbb {R}}^d$ be a bounded open set and denote

59 $$\begin{aligned} \gamma (\Omega ):= \inf \left\{ \frac{\int _\Omega \left|\nabla u \right|^2 + u^2 \, \mathrm {d}{\mathbf {x}}}{\int _{\partial \Omega } u^2 \, \mathrm {d}A} : u \in \mathrm {H}^1(\Omega ), u\big |_{\partial \Omega } \not \equiv 0 \right\} . \end{aligned}$$

Then, the following inequality holds as $\varepsilon \rightarrow 0$:

60 $$\begin{aligned} \gamma (\varepsilon \Omega ) \geqq \varepsilon \gamma (\Omega ). \end{aligned}$$

#### Proof

Consider $u \in \mathrm {H}^1(\varepsilon \Omega )$, $u\big |_{\partial \varepsilon \Omega } \not \equiv 0$. Then, with the change of variable ${\mathbf {y}}= \varepsilon ^{-1} {\mathbf {x}}$,

61 $$\begin{aligned} \int _{\varepsilon \Omega } \left|\nabla u({\mathbf {x}}) \right|^2 + u({\mathbf {x}})^2 \, \mathrm {d}{\mathbf {x}}&= \varepsilon ^{d} \int _{\Omega } \varepsilon ^{-2} \left|\nabla u({\mathbf {y}}) \right|^2 + u({\mathbf {y}})^2 \, \mathrm {d}{\mathbf {y}}\\&\geqq \varepsilon ^{d} \int _{\Omega } \gamma (\Omega ) \int _{\partial \Omega } u({\mathbf {y}})^2 \, \mathrm {d}A\\&= \varepsilon \gamma (\Omega ) \int _{\partial \varepsilon \Omega } u({\mathbf {x}})^2 \, \mathrm {d}A. \end{aligned}$$

$$\square $$

### Uniform bounds on Steklov eigenfunctions

In order to obtain convergence of the eigenfunctions we will need that their $\mathrm {L}^\infty$ norm stays bounded. In this subsection, since we will need to understand specific interplay between boundary surface area and volume, we will diverge from our convention and denote the area of a boundary $\partial \Omega$ by ${\mathcal {H}}^{d-1}(\partial \Omega )$. Let $\mathrm {B}\mathrm {V}(\Omega )$ be the set of functions of bounded variation in $\Omega$, that is the set of $u \in \mathrm {L}^1(\Omega )$ whose derivative *Du* in the sense of distributions is a finite signed Radon measure on $\Omega$. An important feature of that space in our setting is that for bounded subsets $E \subset {\mathbb {R}}^d$ with Lipschitz boundary, their indicator function ${\varvec{1}}_E$ has bounded variation, and $\left|D{\varvec{1}}_E \right|$ is the boundary measure of *E*. In [5, Theorem 3.1], the authors prove that for any Steklov eigenfunction *u* of a domain $\Omega$ with eigenvalue $\sigma$,

62 $$\begin{aligned} \left\| u \right\| _{\mathrm {L}^\infty (\Omega )} \leqq C \left\| u \right\| _{\mathrm {L}^2(\partial \Omega )} \end{aligned}$$

where *C* depends continuously on the dimension *d*, $\sigma$, $\left|\Omega \right|$ and the norm of the trace application

63 $$\begin{aligned} T : \mathrm {B}\mathrm {V}(\Omega ) \rightarrow \mathrm {L}^1(\partial \Omega ). \end{aligned}$$

It is clear that in our situation, the dimension and $\left|\Omega \right|$ stay bounded. The eigenvalues will be shown later to also stay bounded, but we use to control the norm of *T*. In [5, Proposition 5.1], they give the following condition under which said norm stays bounded for a family of domains. If $\left\{ \Omega ^\varepsilon \right\}$ is a family of open bounded domains with ${\mathcal {H}}^{d-1}(\partial \Omega ^\varepsilon ) < \infty$ and such that $\Omega ^\varepsilon \subset K$ for some bounded set *K*, and if there exists constants *Q* and $\delta$ such that for every $x \in \partial \Omega ^\varepsilon$

64 $$\begin{aligned} \sup \left\{ \frac{{\mathcal {H}}^{d-1}(\partial ^* E \cap \partial ^* \Omega ^\varepsilon )}{{\mathcal {H}}^{d-1}(\partial ^* E \cap \Omega ^\varepsilon )} : E \subset \Omega ^\varepsilon \cap B_\delta (x), {\text {Per}}(E,\Omega _n) < \infty \right\} \leqq Q, \end{aligned}$$

then the norm of $T : \mathrm {B}\mathrm {V}(\Omega ^\varepsilon ) \rightarrow \mathrm {L}^1(\partial \Omega ^\varepsilon )$ is uniformly bounded in $\varepsilon$. Here, $\partial ^* E$ denotes the reduced bondary of *E*, which is given (see [23, Definition 3.3]) by the set of $x \in \partial E$ such that

- for all $$r > 0$$, $$\int _{B(x,r)} \left|D{\varvec{1}}_E \right| > 0$$,
- the limit
  $$\begin{aligned} \nu (x) = \lim _{r \searrow 0} \frac{\int _{B(x,r)} D{\varvec{1}}_E}{\int _{B(x,r)} \left|D {\varvec{1}}_E \right|} \end{aligned}$$
  exists, and
- $$\left|\nu (x) \right| = 1$$.

In general, the reduced boundary may be much smaller than the topological boundary but for sets with $C^1$ boundary they coincide.

The next lemma is inspired by [5, Example 2]. Note that in their example, $r_\varepsilon = o_{}\left( \varepsilon ^{\frac{2d-1}{d-1}} \right)$, which means that the radius of the holes if one order of magnitude in $\varepsilon$ smaller than the critical level at which our holes are going to 0. Parts of the proof are in a similar spirit but we need more precise estimates separately around every boundary component.

#### Lemma 16

For all $\varepsilon >0$ sufficiently small, the norm of $T : \mathrm {B}\mathrm {V}(\Omega ^\varepsilon ) \rightarrow \mathrm {L}^1(\partial \Omega ^\varepsilon )$ is uniformly bounded in $\varepsilon$.

#### Proof

Following (66), for any $x_\varepsilon \in \partial \Omega ^\varepsilon$, we want to give a uniform upper bound for the ratio

65 $$\begin{aligned} \frac{{\mathcal {H}}^{d-1}(\partial ^* E \cap \partial \Omega ^\varepsilon )}{{\mathcal {H}}^{d-1}(\partial ^* E \cap \Omega ^\varepsilon )} \end{aligned}$$

for sets $E \subset B_\delta (x_\varepsilon ) \cap \Omega ^\varepsilon$. Let us make the observation that for $\varepsilon >0$ small enough,

66 $$\begin{aligned} \#\left\{ {\mathbf {n}}\in I^\varepsilon : Q_{\mathbf {n}}^\varepsilon \cap B_\delta (x_\varepsilon ) \ne \emptyset \right\} \leqq 2\omega _d\left( \frac{\delta }{\varepsilon }\right) ^d, \end{aligned}$$

and for some *M*, ${\mathcal {H}}^{d-1}(\partial \Omega \cap \overline{B_\delta (x_\varepsilon )}) \leqq M\delta ^{d-1}$. On one hand, setting ${\widetilde{\beta }} = \max (\beta ,1)$, for any $E \subset \Omega ^\varepsilon \cap B_\delta (x_\varepsilon )$ of finite perimeter,

67 $$\begin{aligned} {\mathcal {H}}^{d-1}(\partial ^* E \cap \partial \Omega ^\varepsilon ) \leqq {\mathcal {H}}^{d-1}(\partial \Omega ^\varepsilon \cap \overline{B_\delta (x_\varepsilon )}) \leqq \left( M + 2d{\widetilde{\beta }} \delta \right) \delta ^{d-1}. \end{aligned}$$

On the other hand, we now need to find a lower bound for the denominator in (67) terms of the numerator. By definition of $I^\varepsilon$, for all ${\mathbf {n}}\in I^\varepsilon$ we have that $\partial \Omega ^\varepsilon \cap Q_{\mathbf {n}}^\varepsilon = \partial T_{\mathbf {n}}^\varepsilon$. From this, we can decompose

68 $$\begin{aligned} {\mathcal {H}}^{d-1}(\partial ^*E \cap \partial \Omega ^\varepsilon ) = {\mathcal {H}}^{d-1}(\partial ^* E \cap \partial \Omega ) + \sum _{{\mathbf {n}}\in I^\varepsilon } {\mathcal {H}}^{d-1}(\partial ^*E \cap \partial T_{\mathbf {n}}^\varepsilon ). \end{aligned}$$

We first observe that if $\delta$ is chosen such that $\omega _d \delta ^d \leqq \left|\Omega \right|/2$ and ${\mathcal {H}}^d(E)\leqq 1$, the trace inequality for $BV(\Omega )\rightarrow L^1(\partial \Omega )$ and the relative isoperimetric inequality relative to $\Omega$ [5, Inequality (2.2)] applied to the characteristic function $\chi _E\in BV(\Omega )$ implies that there is a constant *C*, whose precise value may change from line to line but which depens only on $\Omega$, such that

69 $$\begin{aligned} \begin{aligned} {\mathcal {H}}^{d-1}(\partial ^*E \cap \partial \Omega )&\leqq C {\mathcal {H}}^{d-1}(\partial ^*E \cap \Omega )\\&= C ({\mathcal {H}}^{d-1}(\partial ^*E \cap (\partial \Omega ^\varepsilon {\setminus } \partial \Omega )) + {\mathcal {H}}^{d-1}(\partial ^*E \cap \Omega ^\varepsilon ))\\&= C(\sum _{{\mathbf {n}}\in I^\varepsilon } {\mathcal {H}}^{d-1}(\partial ^*E \cap \partial T_{\mathbf {n}}^\varepsilon ) + {\mathcal {H}}^{d-1}(\partial ^*E \cap \Omega ^\varepsilon )) \end{aligned} \end{aligned}$$

Substitution in (70) implies

70 $$\begin{aligned} {\mathcal {H}}^{d-1}(\partial ^*E \cap \partial \Omega ^\varepsilon ) \leqq C\left( {\mathcal {H}}^{d-1}(\partial ^* E \cap \Omega ^\varepsilon ) + \sum _{{\mathbf {n}}\in I^\varepsilon } {\mathcal {H}}^{d-1}(\partial ^*E \cap \partial T_{\mathbf {n}}^\varepsilon )\right) . \end{aligned}$$

For ${\mathbf {n}}\in I^\varepsilon$ and $t\in (0,\varepsilon /4)$, define

71 $$\begin{aligned} F_{{\mathbf {n}},t} := \{x\in E\,:\,{{\,\mathrm{dist}\,}}_{\partial T_{\mathbf {n}}^\varepsilon }(x) \leqq t\}\subset Q_{\mathbf {n}}^\varepsilon . \end{aligned}$$

Assume that there is $t \in (0,\varepsilon /4)$ such that

72 $$\begin{aligned} {\mathcal {H}}^{d-1}(\partial ^* F_{{\mathbf {n}},t} \cap Q_{{\mathbf {n}}}^\varepsilon ) \leqq 2{\mathcal {H}}^{d-1}(\partial ^* E \cap Q_{\mathbf {n}}^\varepsilon ). \end{aligned}$$

Since projections on convex sets are nonexpansive and $F_{{\mathbf {n}},t} \subset Q_{{\mathbf {n}}}^\varepsilon$, we have that

73 $$\begin{aligned} \begin{aligned} {\mathcal {H}}^{d-1}(\partial ^* E \cap \partial T_{\mathbf {n}}^\varepsilon )&= {\mathcal {H}}^{d-1}(\partial ^* F_{{\mathbf {n}},t} \cap \partial T_{\mathbf {n}}^\varepsilon ) \\&\leqq {\mathcal {H}}^{d-1}(\partial ^* F_{{\mathbf {n}},t} \cap Q_{{\mathbf {n}}}^\varepsilon ) \\&\leqq 2{\mathcal {H}}^{d-1}(\partial ^*E \cap Q_{{\mathbf {n}}}^\varepsilon ). \end{aligned} \end{aligned}$$

If (74) holds for each ${\mathbf {n}}\in I^\varepsilon$ then it follows from (72) that

74 $$\begin{aligned} \begin{aligned} {\mathcal {H}}^{d-1}(\partial ^*E \cap \partial \Omega ^\varepsilon )&\leqq C\left( {\mathcal {H}}^{d-1}(\partial ^* E \cap \Omega ^\varepsilon ) + \sum _{{\mathbf {n}}\in I^\varepsilon }{\mathcal {H}}^{d-1}(\partial ^*E \cap Q_{{\mathbf {n}}}^\varepsilon ) \right) \\&\leqq C{\mathcal {H}}^{d-1}(\partial ^* E \cap \Omega ^\varepsilon ), \end{aligned} \end{aligned}$$

which completes the proof in this case.

Otherwise, let $J^\varepsilon := \left\{ {\mathbf {n}}\in I^\varepsilon : \text {equation } (28) \text { does not hold} \right\}$. For ${\mathbf {n}}\in J^\varepsilon$, set

75 $$\begin{aligned} h_{\mathbf {n}}(t) := {\mathcal {H}}^{d-1}\left( \{x\in \partial ^*F_{{\mathbf {n}},t}\,:\, {{\,\mathrm{dist}\,}}_{\partial T_{\mathbf {n}}^\varepsilon }(x) = t\}\right) . \end{aligned}$$

Since (74) does not hold,

$$\begin{aligned} 2h_{\mathbf {n}}(t)\geqq {\mathcal {H}}^{d-1}(\partial ^*F_{{\mathbf {n}},t} \cap Q_{\mathbf {n}}^\varepsilon ). \end{aligned}$$

It follows from the relative isoperimetric inequality with respect to $Q_{\mathbf {n}}^\varepsilon$ that

76 $$\begin{aligned} \begin{aligned} c{\mathcal {H}}^d(F_{{\mathbf {n}},t})^{\frac{d-1}{d}}&\leqq {\mathcal {H}}^{d-1}(\partial ^*F_{{\mathbf {n}},t} \cap Q_{\mathbf {n}}^\varepsilon ) \leqq 2 h_{\mathbf {n}}(t). \end{aligned} \end{aligned}$$

The coarea formula gives $\partial _t {\mathcal {H}}^{d}(F_{{\mathbf {n}},t}) = h_{\mathbf {n}}(t)$, and it follows by integration and the relative isoperimetric inequality with respect to $Q_{\mathbf {n}}^\varepsilon$ that

77 $$\begin{aligned} \begin{aligned} d{\mathcal {H}}^d(F_{{\mathbf {n}},\varepsilon /4})^{1/d}&= \int _0^{\varepsilon /4} \frac{h_{\mathbf {n}}(t)}{{\mathcal {H}}^{d}(F_{{\mathbf {n}},t})^{\frac{d-1}{d}}} \, \mathrm {d}t \geqq C \varepsilon . \end{aligned} \end{aligned}$$

That is for ${\mathbf {n}}\in J^\varepsilon$,

78 $$\begin{aligned} \begin{aligned} {\mathcal {H}}^d(E\cap Q_{\mathbf {n}}^\varepsilon )&\geqq {\mathcal {H}}^d(F_{{\mathbf {n}},\varepsilon /4})\\&\geqq C\varepsilon ^d\\&={\mathcal {H}}^d(Q_{\mathbf {n}}^\varepsilon ). \end{aligned} \end{aligned}$$

This implies that for $\varepsilon >0$ small enough one has

79 $$\begin{aligned} \begin{aligned} {\mathcal {H}}^d(E\cap Q_{\mathbf {n}}^\varepsilon )&\geqq \frac{C}{{\widetilde{\beta }}}{\mathcal {H}}^{d-1}(\partial T_{\mathbf {n}}^\varepsilon ). \end{aligned} \end{aligned}$$

The isoperimetric inequality for $E\subset {\mathbb {R}}^d$ gives

80 $$\begin{aligned} \begin{aligned} \sum _{{\mathbf {n}}\in J^\varepsilon }{\mathcal {H}}^{d-1}(\partial T_{\mathbf {n}}^\varepsilon )&\leqq {\widetilde{\beta }}\sum _{{\mathbf {n}}\in J^\varepsilon }{\mathcal {H}}^d(E \cap Q_{\mathbf {n}}^\varepsilon )\\&\leqq {\widetilde{\beta }}{\mathcal {H}}^d(E)\\&\leqq C{\widetilde{\beta }} {\mathcal {H}}^{d-1}(\partial ^*E)^{\frac{d}{d-1}}\\&=C{\widetilde{\beta }}\left( {\mathcal {H}}^{d-1}(\partial ^*E \cap \partial \Omega ) + {\mathcal {H}}^{d-1}(\partial ^*E \cap \partial T^\varepsilon ) + {\mathcal {H}}^{d-1}(\partial ^*E \cap \Omega ^\varepsilon )\right) ^{\frac{d}{d-1}}. \end{aligned} \end{aligned}$$

Together with (71) and (75) this leads to the existence of a constant *C* which can depend on $\beta$, such that

81 $$\begin{aligned} \begin{aligned} \sum _{{\mathbf {n}}\in J^\varepsilon }{\mathcal {H}}^{d-1}(\partial T_{\mathbf {n}}^\varepsilon )\leqq C \left( {\mathcal {H}}^{d-1}(\partial ^*E \cap \Omega ^\varepsilon ) + \sum _{{\mathbf {n}}\in J^\varepsilon }{\mathcal {H}}^{d-1}(\partial T_{\mathbf {n}}^\varepsilon )\right) ^{\frac{d}{d-1}}. \end{aligned} \end{aligned}$$

Because $J^\varepsilon \ne \emptyset$, dividing and factoring, this leads to

82 $$\begin{aligned} 1\leqq C\left( \sum _{{\mathbf {n}}\in J^\varepsilon }{\mathcal {H}}^{d-1}(\partial T_{\mathbf {n}}^\varepsilon )\right) ^{\frac{1}{d-1}}\left( \frac{{\mathcal {H}}^{d-1}(\partial ^*E \cap \Omega ^\varepsilon )}{\sum _{{\mathbf {n}}\in J^\varepsilon }{\mathcal {H}}^{d-1}(\partial T_{\mathbf {n}}^\varepsilon )} + 1\right) ^{\frac{d}{d-1}}. \end{aligned}$$

For each ${\mathbf {n}}\in J^\varepsilon$, $Q_{\mathbf {n}}^\varepsilon \subset B_{2\delta }(x_\varepsilon )$ and it follows from (69) that we can choose $\delta$ small enough depending on $\Omega$, the dimension, and $\beta$ but not on $\varepsilon$ such that

83 $$\begin{aligned} C\left( \sum _{{\mathbf {n}}\in J^\varepsilon }{\mathcal {H}}^{d-1}(\partial T_{\mathbf {n}}^\varepsilon )\right) ^{\frac{1}{d-1}}<\frac{1}{4}. \end{aligned}$$

This implies that there is a constant $c>0$ such that for all $\varepsilon > 0$,

84 $$\begin{aligned} 1 \leqq \frac{{\mathcal {H}}^{d-1}(\partial ^*E \cap \Omega ^\varepsilon )}{\sum _{{\mathbf {n}}\in J^\varepsilon } {\mathcal {H}}^{d-1}(\partial T_{\mathbf {n}}^\varepsilon )}. \end{aligned}$$

Combining (72), (75) and (86), provides a constant *C* such that

85 $$\begin{aligned} {\mathcal {H}}^{d-1}(\partial ^*E \cap \partial \Omega ^\varepsilon ) \leqq C {\mathcal {H}}^{d-1}(\partial ^*E \cap \Omega ^\varepsilon ). \end{aligned}$$

$$\square $$

#### Remark 17

When $r_\varepsilon = o_{}\left( \varepsilon ^{\frac{d}{d-1}} \right)$, (that is in the subcritical regime), the previous result along with [5, Theorem 4.1] implies convergence of the eigenvalues of the Steklov problem on $\Omega ^\varepsilon$ to the eigenvalues of the Steklov problem on $\Omega$.

## Homogenisation of the Steklov problem

Let us first establish some basic facts related to the geometry of the homogenisation problem, under the assumption that $r_\varepsilon ^{d-1} \varepsilon ^{-d} \rightarrow \beta \in [0,\infty )$ as $\varepsilon \rightarrow 0$. The number of holes $N(\varepsilon )=\#I^\varepsilon$ satisfies

$$\begin{aligned} N(\varepsilon ) \sim |\Omega |\varepsilon ^{-d} \qquad \text{ as } \varepsilon \rightarrow 0. \end{aligned}$$

This implies that

86 $$\begin{aligned} \left|T^\varepsilon \right| = \sum _{{\mathbf {k}}\in I^\varepsilon }|T_{{\mathbf {k}}}^\varepsilon | = O_{}\left( r_\varepsilon \right) \qquad \text{ and }\qquad \left|\partial T^\varepsilon \right|= \sum _{{\mathbf {k}}\in I^\varepsilon }|\partial T_{{\mathbf {k}}}^\varepsilon |\sim A_d \beta |\Omega |. \end{aligned}$$

The remainder of the section is split into three parts. In the first one we extend the functions $u_k^\varepsilon$ to the whole of $\Omega$, in order to obtain weak $\mathrm {H}^1$ convergence, up to taking subsequences. In the second part, we prove that those converging subsequences converge to solutions of Problem (4). Finally, we prove in the third part that the only functions they can converge to are the corresponding eigenfunction in (4), implying convergence as $\varepsilon \rightarrow 0$, with this understood in the sense of Remark 3 if the limit problem has eigenvalues that are not simple.

### Extension of eigenfunctions

For $k \geqq 1$, recall that $u_k^{\varepsilon }:\Omega ^\varepsilon \rightarrow {\mathbb {R}}$ is the *k*’th Steklov eigenfunction on $\Omega ^\varepsilon$. Then

$$\begin{aligned} {\left\{ \begin{array}{ll} \Delta u_k^{\varepsilon }=0&{} \text{ in } \Omega ^\varepsilon ,\\ \partial _{\nu }u_k^{\varepsilon }=\sigma _k^{\varepsilon }u_k^{\varepsilon }&{} \text{ on } \partial \Omega ^\varepsilon . \end{array}\right. } \end{aligned}$$

Recall also that the eigenfunctions $u_k^{\varepsilon }$ are normalized by requiring that

$$\begin{aligned} \int _{\partial \Omega ^\varepsilon }\left( u_k^{\varepsilon }\right) ^2 \, \mathrm {d}A=1. \end{aligned}$$

Define the function $U_k^{\varepsilon }\in \mathrm {H}^1(\Omega )$ to be the harmonic extension of $u_k^{\varepsilon }$ to the interior of the holes, so

$$\begin{aligned} {\left\{ \begin{array}{ll} U_k^{\varepsilon }=u_k^{\varepsilon }&{} \text{ in } \overline{\Omega ^\varepsilon },\\ \Delta U_k^{\varepsilon }=0&{} \text{ in } T^\varepsilon . \end{array}\right. } \end{aligned}$$

#### Lemma 18

There is a sequence $\varepsilon _n \rightarrow 0$ such that $U_k^{\varepsilon _n}$ has a weak limit in $\mathrm {H}^1(\Omega )$.

#### Proof

It suffices to show that $\left\{ U_k^\varepsilon : 0 <\varepsilon \leqq 1 \right\}$ is bounded in $\mathrm {H}^1(\Omega )$. Recall that

87 $$\begin{aligned} \left\| U_k^{\varepsilon } \right\| ^2_{H^1} = \left\| U_k^{\varepsilon } \right\| ^2_{\mathrm {L}^2} + {\mathcal {D}}\left( U_k^{\varepsilon }\right) . \end{aligned}$$

We first bound the $\mathrm {L}^2$ norm of $U_{k}^{\varepsilon }$. Let $\lambda$ be the first eigenvalue of the following Robin problem on $\Omega$:

$$\begin{aligned} {\left\{ \begin{array}{ll} -\Delta u= \lambda u&{} \text{ in } \Omega ,\\ \partial _\nu u= - u &{} \text{ on } \partial \Omega . \end{array}\right. } \end{aligned}$$

It is well known (see *for example* [4]) that $\lambda >0$ and that it admits the following characterization:

$$\begin{aligned} \lambda =\inf _{v \in \mathrm {H}^1(\Omega )} \frac{\int _\Omega |\nabla v|^2 \, \mathrm {d}{\mathbf {x}}+\int _{\partial \Omega }v^2\, \mathrm {d}S}{\int _{\Omega }v^2\, \mathrm {d}{\mathbf {x}}}. \end{aligned}$$

Applying this to $v=U_k^{\varepsilon }$ leads to

88 $$\begin{aligned} \begin{aligned} \int _\Omega \left( U_k^{\varepsilon }\right) ^2 \, \mathrm {d}{\mathbf {x}}&\leqq \frac{1}{\lambda }\left( \int _\Omega \left| \nabla U_k^{\varepsilon }\right| ^2 \, \mathrm {d}{\mathbf {x}}+\int _{\partial \Omega }\left( U_k^{\varepsilon }\right) ^2 \, \mathrm {d}A\right) \\&\leqq \frac{1}{\lambda }\left( {\mathcal {D}}\left( U_k^{\varepsilon }\right) +1\right) . \end{aligned} \end{aligned}$$

It is therefore sufficient to bound the Dirichlet energy. We first see that

89 $$\begin{aligned} {\mathcal {D}}\left( U_k^{\varepsilon };\Omega \right) = {\mathcal {D}}\left( u_k^{\varepsilon };\Omega ^\varepsilon \right) + {\mathcal {D}}\left( U_k^{\varepsilon };T^\varepsilon \right) =\sigma _k^\varepsilon + {\mathcal {D}}\left( U_k^{\varepsilon };T^\varepsilon \right) . \end{aligned}$$

It follows from Lemma 12 and monotonicity of the Dirichlet energy, that the contribution from the holes is

90 $$\begin{aligned} \begin{aligned} {\mathcal {D}}\left( U_k^{\varepsilon };T^\varepsilon \right)&=\sum _{{\mathbf {k}}\in I^\varepsilon }{\mathcal {D}}\left( U_{k}^{\varepsilon };T_{{\mathbf {k}}}^\varepsilon \right) \\&\leqq \sum _{{\mathbf {k}}\in I^\varepsilon } 5\left( \frac{r_\varepsilon }{\varepsilon }\right) ^d {\mathcal {D}}\left( u_k^{\varepsilon };Q_{{\mathbf {k}}}^\varepsilon \right) \left( 1 + O_{}\left( \left( \frac{ r_\varepsilon }{\varepsilon } \right) ^d \right) \right) \\&\leqq 5 \left( \frac{r_\varepsilon }{\varepsilon }\right) ^d {\mathcal {D}}\left( u_k^{\varepsilon };\Omega ^\varepsilon \right) \left( 1 + O_{}\left( \left( \frac{r_\varepsilon }{\varepsilon } \right) ^d \right) \right) \\&\leqq C \sigma _k(\Omega ^\varepsilon ), \end{aligned} \end{aligned}$$

for some constant *C*. Combining (92) and (90), we see that, to bound $\left\| U_k^\varepsilon \right\| _{\mathrm {H}^1(\Omega )}$, it is sufficient to find a bound for $\sigma _k^\varepsilon$ independent of $\varepsilon$. The variational characterisation for Steklov eigenvalues can be rewritten as

91 $$\begin{aligned} \sigma _k^\varepsilon = \min _{\begin{array}{c} E \subset \mathrm {L}^2(\partial \Omega ^\varepsilon ) \\ \dim (E) = k+1 \end{array}} \max _{u \in E} \frac{{\mathcal {D}}(u)}{\left\| u \right\| ^2_{\mathrm {L}^2(\partial \Omega ^\varepsilon )}}. \end{aligned}$$

We use eigenfunctions of the dynamical eigenvalue problem as a test subspace for $\sigma _k^\varepsilon$. Namely, setting $E = {\text {span}}(U_0,\dotsc ,U_{k})$ we see that for $\varepsilon$ small enough it spans a $k+1$ dimensional subspace of $\mathrm {L}^2(\partial \Omega ^\varepsilon )$. Indeed, they are an orthonormal set with respect to $(\cdot ,\cdot )_\beta$, and for every $0 \leqq j,\ell \leqq k$,

92 $$\begin{aligned} (U_j,U_\ell )_{\partial ^\varepsilon } \xrightarrow {\varepsilon \rightarrow 0} (U_j,U_\ell )_{\beta }. \end{aligned}$$

Therefore, from the characterisation of the eigenvalues $\Sigma _{k,\beta }$,

93 $$\begin{aligned} \begin{aligned} \sigma _k^\varepsilon&\leqq \max _{U \in E} \frac{{\mathcal {D}}(U)}{(U,U)_{\partial ^\varepsilon }} \\&\leqq \Sigma _{k,\beta } + o_{}\left( 1 \right) . \end{aligned} \end{aligned}$$

This completes the proof that $\left\{ U_k^\varepsilon \right\}$ is bounded so that there is a converging subsequence as $\varepsilon \rightarrow 0$. $\square$

From now on we will abuse notation and relabel that sequence $\varepsilon _n \rightarrow 0$ along which $U_k^\varepsilon$ has a weak limit as $\varepsilon \rightarrow 0$ again.

### Establishing the limit problem

Our aim by the end of this subsection is to prove the following weaker version of Theorem 2:

#### Proposition 19

Let $k\in {\mathbb {N}}$. As $\varepsilon \rightarrow 0$, the pairs $\left( \sigma _k^\varepsilon ,U_k^\varepsilon \right)$ converge to a solution $(\Sigma ,U)$ of (4), the convergence of the functions $U_k^\varepsilon$ being weak in $\mathrm {H}^1(\Omega )$.

Up to choosing a subsequence, we assume that $\sigma _k^{\varepsilon }$ converges to some number $\Sigma$ and also that $\left\{ U_k^{\varepsilon } \right\} \subset \mathrm {H}^1(\Omega )$ is weakly converging in $\mathrm {H}^1(\Omega )$ to some $U \in \mathrm {H}^1(\Omega )$, from which we also get strong convergence to *U* in $\mathrm {L}^2(\Omega )$. Considering the real-valued test function $V\in \mathrm {H}^1(\Omega )$, we see that

$$\begin{aligned} \int _\Omega \nabla U_k^{\varepsilon }\cdot \nabla V \, \mathrm {d}{\mathbf {x}}&= \int _{\Omega ^\varepsilon } \nabla u_k^{\varepsilon }\cdot \nabla V \, \mathrm {d}{\mathbf {x}}+\int _{T^\varepsilon } \nabla U_k^{\varepsilon }\cdot \nabla V \, \mathrm {d}{\mathbf {x}}\\&=\sigma _k^{\varepsilon }\int _{\partial \Omega ^\varepsilon }u_k^{\varepsilon }V \, \mathrm {d}{\mathbf {x}}+\int _{T^\varepsilon } \nabla U_k^{\varepsilon }\cdot \nabla V \, \mathrm {d}{\mathbf {x}}\\&=\sigma _k^{\varepsilon }\int _{\partial \Omega }u_k^{\varepsilon }V \, \mathrm {d}A+\sigma _k^{\varepsilon }\int _{\partial T^\varepsilon }u_k^{\varepsilon }V \, \mathrm {d}A +\int _{T^\varepsilon } \nabla U_k^{\varepsilon }\cdot \nabla V \, \mathrm {d}{\mathbf {x}}. \end{aligned}$$

Letting $\varepsilon \rightarrow 0$ leads, if the limits exist, to

$$\begin{aligned} \int _\Omega \nabla U\cdot \nabla V \, \mathrm {d}{\mathbf {x}}-\Sigma \int _{\partial \Omega }UV \, \mathrm {d}A= \Sigma \lim _{\varepsilon \rightarrow 0} \int _{\partial T^\varepsilon }u_k^{\varepsilon } V \, \mathrm {d}A+\lim _{\varepsilon \rightarrow 0}\int _{T^\varepsilon } \nabla U_k^{\varepsilon }\cdot \nabla V \, \mathrm {d}{\mathbf {x}}. \end{aligned}$$

It follows from the Cauchy–Schwarz inequality that

$$\begin{aligned} \int _{T^\varepsilon } \nabla U_k^{\varepsilon }\cdot \nabla V \, \mathrm {d}{\mathbf {x}}\leqq \left( \int _{T^\varepsilon } \left| \nabla U_k^{\varepsilon }\right| ^2 \, \mathrm {d}{\mathbf {x}}\int _{T^\varepsilon }|\nabla V|^2 \, \mathrm {d}{\mathbf {x}}\right) ^{1/2}, \end{aligned}$$

which tends to 0 according to Lemma 12. It follows that

$$\begin{aligned} \int _\Omega \nabla U\cdot \nabla V \, \mathrm {d}{\mathbf {x}}-\Sigma \int _{\partial \Omega }UV \, \mathrm {d}A= \Sigma \lim _{\varepsilon \rightarrow 0}\int _{\partial T^\varepsilon }u_k^{\varepsilon }V \, \mathrm {d}A, \end{aligned}$$

and all that is left to do is to analyse the last term.

#### Proposition 20

Suppose that $\varepsilon ^{-d}r_\varepsilon ^{d-1}\rightarrow \beta \geqq 0$. Then, for each $V\in \mathrm {H}^1(\Omega )$, the following holds:

94 $$\begin{aligned} \lim _{\varepsilon \rightarrow 0}\int _{\partial T^\varepsilon }u_k^{\varepsilon }V \, \mathrm {d}A= A_d\beta \int _{\Omega }UV \, \mathrm {d}{\mathbf {x}}. \end{aligned}$$

#### Remark 21

The functional $V\mapsto \int _{\partial T^\varepsilon }u_k^{\varepsilon }V$ is bounded on $H^1(\Omega )$. By the Riesz–Fréchet representation theorem, there exists a function $\xi ^\varepsilon \in H^1(\Omega )$ such that

$$\begin{aligned} \int _{\partial T^\varepsilon }u_k^{\varepsilon }V =\int _{\Omega }\nabla \xi ^\varepsilon \cdot \nabla V+\xi ^\varepsilon V\,\mathrm{d}x\qquad \forall V\in H^1(\Omega ). \end{aligned}$$

Using appropriate test functions shows that $\xi ^\varepsilon$ is the weak solution of the following transmission problem:

95 $$\begin{aligned} {\left\{ \begin{array}{ll} \Delta \xi ^\varepsilon =0&{} \text{ in } \Omega ^\varepsilon \cup T^\varepsilon ,\\ \partial _\nu \xi ^\varepsilon _++\partial _\nu \xi ^\varepsilon _-=u_k^\varepsilon &{} \text{ on } \partial T^\varepsilon ,\\ \partial _\nu \xi ^\varepsilon =0&{} \text{ on } \partial \Omega . \end{array}\right. } \end{aligned}$$

Proposition 20 is an homogenisation result for this problem. It means that in the limit as $\varepsilon \rightarrow 0$, the solution converges to that of the following problem:

96 $$\begin{aligned} {\left\{ \begin{array}{ll} -\Delta \Xi +(1-A_d\beta )\Xi =0&{} \text{ in } \Omega ,\\ \partial _\nu \Xi =0&{} \text{ on } \partial \Omega . \end{array}\right. } \end{aligned}$$

Transmission problems have recently been the subject of investigation through means of homogenisation, see for example [35].

The proof of Proposition 20 is divided in three main steps. In the first step, we justify that we can use smooth test functions in the limit (96). In order to do so, we will use an inner representation of the lefthandside in (96), defined in terms of extensions to the holes, to show that it is bounded, uniformly in $\varepsilon$. In this representation, however, it is hard to explicitly compute the limit problem.

In our second step, we introduce an outer representation of the lefthandside in (96), through integration on the outside of the holes. This representation is given in terms of an auxilliary function $\Psi$, for which we derive some regularity properties.

In the final step, we use this latter representation to show that the limit (96) indeed holds. Here, we reap rewards from the previous steps and use explicitly the properties of the auxilliary function $\Psi$, as well as better estimates awarded from the fact that we can test against smooth functions.

#### Proof of Proposition 20

Define the family of bounded functionals $L_\varepsilon :\mathrm {H}^1(\Omega )\rightarrow {\mathbb {R}}$ by

$$\begin{aligned} L_\varepsilon (V):=\int _{\partial T_\varepsilon } V \, \mathrm {d}A. \end{aligned}$$

**Step 1**: Inner representation of $L_\varepsilon$.

Define $$\varphi _\varepsilon :{\mathbb {R}}^d\rightarrow {\mathbb {R}}$$ by

97 $$\begin{aligned} \varphi _\varepsilon ({\mathbf {x}}) = {\left\{ \begin{array}{ll} \frac{ \left|{\mathbf {x}} \right|^2}{2r_\varepsilon }&{}\text {for }x\in B(0,r_\varepsilon ),\\ 0&{}\text {elsewhere}. \end{array}\right. } \end{aligned}$$

By periodizing along $\varepsilon {\mathbb {Z}}^d$ we obtain the function $\Phi _\varepsilon : {\mathbb {R}}^d \rightarrow {\mathbb {R}}$ given by

98 $$\begin{aligned} \Phi _\varepsilon ({\mathbf {x}}) := \sum _{{\mathbf {k}}\in I^\varepsilon } \varphi _\varepsilon ({\mathbf {x}}- \varepsilon {\mathbf {k}}). \end{aligned}$$

#### Lemma 22

The functional $L_\varepsilon :\mathrm {H}^1(\Omega )\rightarrow {\mathbb {R}}$ admits the following representation:

99 $$\begin{aligned} L_\varepsilon (V) = \frac{d}{r_\varepsilon } \int _{T^\varepsilon } V \, \mathrm {d}{\mathbf {x}}+ \int _{T^\varepsilon } \nabla \Phi _\varepsilon \cdot \nabla V \, \mathrm {d}{\mathbf {x}}. \end{aligned}$$

#### Proof

It is straightforward to check that

100 $$\begin{aligned} {\left\{ \begin{array}{ll} \Delta \Phi _\varepsilon = \frac{d}{r_\varepsilon } &{} \text {in } T^\varepsilon ,\\ \partial _\nu \Phi _\varepsilon = 1 &{} \text {on } \partial T^\varepsilon ,\\ \Phi = 0 &{} \text {in } \Omega ^\varepsilon . \end{array}\right. } \end{aligned}$$

The function $\Phi _\varepsilon$ therefore satisfies the weak identity

101 $$\begin{aligned} \int _{T^\varepsilon } \nabla \Phi _\varepsilon \cdot V = - \frac{d}{r_\varepsilon } \int _{T^\varepsilon } V + \int _{\partial T^\varepsilon } V,\qquad \forall V\in H^1(\Omega ). \end{aligned}$$

$$\square $$

For each $k\in {\mathbb {N}}$ and $\varepsilon >0$, the functional $\widetilde{L_\varepsilon }:H^1(\Omega )\rightarrow {\mathbb {R}}$ is defined by

$$\begin{aligned} \widetilde{L_\varepsilon }(V):=L_\varepsilon (u_k^{\varepsilon }V). \end{aligned}$$

#### Lemma 23

There is an $\varepsilon _0>0$ such that the family $\left\{ \widetilde{L_\varepsilon }\right\} _{\varepsilon >0}\subset \left( H^1(\Omega )\right) ^*$ is uniformly bounded for $0 < \varepsilon \leqq \varepsilon _0$.

#### Proof

Given $V \in \mathrm {H}^1(\Omega )$, it follows from the Lemma 22 that

102 $$\begin{aligned} \begin{aligned} L_\varepsilon (U_k^\varepsilon V) = \frac{d}{r_\varepsilon } \int _{T^\varepsilon } U_k^\varepsilon V \, \mathrm {d}{\mathbf {x}}+ \int _{T^\varepsilon } \nabla \Phi _\varepsilon \cdot \nabla (U_k^\varepsilon V) \, \mathrm {d}{\mathbf {x}}. \end{aligned} \end{aligned}$$

To bound the first term, start by using the Cauchy–Schwarz inequality to get that

103 $$\begin{aligned} \left|\frac{d}{r_\varepsilon }\int _{T^\varepsilon } U_k^\varepsilon V \, \mathrm {d}{\mathbf {x}} \right| \leqq \frac{d}{r_\varepsilon }\left\| U_k^\varepsilon \right\| _{\mathrm {L}^2(T^\varepsilon )} \left\| V \right\| _{\mathrm {L}^2(T^\varepsilon )}. \end{aligned}$$

It follows from Lemma 14 that there is $C > 0$ depending only on $\beta$ such that

104 $$\begin{aligned} \begin{aligned} \left\| V \right\| _{\mathrm {L}^2(T^\varepsilon )}&\leqq C r_\varepsilon ^{1/2} \left\| V \right\| _{\mathrm {H}^1(\Omega )},\\ \left\| U_{k}^\varepsilon \right\| _{\mathrm {L}^2(T^\varepsilon )}&\leqq C r_\varepsilon ^{1/2} \left\| U_k^\varepsilon \right\| _{\mathrm {H}^1(\Omega )}, \end{aligned} \end{aligned}$$

so that

105 $$\begin{aligned} \sup _{\varepsilon \in (0,1)} \left|\frac{d}{r_\varepsilon } \int _{T^\varepsilon }U_k^\varepsilon V \, \mathrm {d}{\mathbf {x}} \right| < \infty . \end{aligned}$$

To bound the second term in (104), the generalised Hölder inequality leads to

$$\begin{aligned} \begin{aligned} \left|\int _{T^\varepsilon } \nabla \Phi _\varepsilon \cdot \nabla (U_k^\varepsilon V) \, \mathrm {d}{\mathbf {x}} \right|&\leqq \left|\int _{T^\varepsilon } U_k^\varepsilon \nabla \Phi _\varepsilon \cdot \nabla V + V \nabla \Phi _\varepsilon \cdot \nabla U_k^\varepsilon \, \mathrm {d}{\mathbf {x}} \right| \\&\leqq \left\| U_k^\varepsilon \right\| _{\mathrm {L}^2(T^\varepsilon )} \left\| \nabla V \right\| _{\mathrm {L}^2(T^\varepsilon )}\left\| \nabla \Phi _\varepsilon \right\| _{\mathrm {L}^\infty (T^\varepsilon )}\\&\qquad + \left\| \nabla U_k^\varepsilon \right\| _{\mathrm {L}^2(T^\varepsilon )} \left\| V \right\| _{\mathrm {L}^2(T^\varepsilon )} \left\| \nabla \Phi _\varepsilon \right\| _{\mathrm {L}^\infty (T^\varepsilon )}\\&\leqq \left( \left\| U_k^\varepsilon \right\| _{\mathrm {L}^2(T^\varepsilon )}+\left\| \nabla U_k^\varepsilon \right\| _{\mathrm {L}^2(T^\varepsilon )}\right) \left\| \nabla V \right\| _{H^1(\Omega )}\left\| \nabla \Phi _\varepsilon \right\| _{\mathrm {L}^\infty (T^\varepsilon )}\\&\leqq \left\| U_k^\varepsilon \right\| _{H^1(T^\varepsilon )}\left\| \nabla V \right\| _{H^1(\Omega )}. \end{aligned} \end{aligned}$$

In the last inequality we have used $\left\| \nabla \Phi _\varepsilon \right\| _{\mathrm {L}^\infty (T^\varepsilon )}=1$, which follows from (99). This quantity is uniformly bounded as $\varepsilon \searrow 0$ since we have shown in the proof of Lemma 18 that $U_k^\varepsilon$ is bounded in $H^1(\Omega )$. Together with (107) this proves for each $V\in H^1(\Omega )$ the existence of a constant *C* such that $|L_\varepsilon (U_k^\varepsilon V)|\leqq C\Vert V\Vert$ for each $\varepsilon$, and the conclusion follows from the Banach–Steinhaus theorem. $\square$

**Step 2**: Outer representation of $L_\varepsilon$. Consider the torus ${\mathcal {C}}={\mathbb {T}}^d = {\mathbb {R}}^d / {\mathbb {Z}}^d$ and introduce the fundamental cell ${\mathcal {C}}^\varepsilon$ as the perforated torus

$$\begin{aligned} {\mathcal {C}}^\varepsilon :={\mathcal {C}}{\setminus } \overline{B\left( 0,\rho _\varepsilon \right) }, \end{aligned}$$

where $\rho _\varepsilon := \varepsilon ^{-1} r_\varepsilon$ is the renormalised radius. Following [41], we define the function $\psi _\varepsilon \in \mathrm {H}^1({\mathcal {C}}^\varepsilon )$ through the weak variational problem

106 $$\begin{aligned} \int _{{\mathcal {C}}^\varepsilon }\nabla \psi _\varepsilon \cdot \nabla V=-c_\varepsilon \int _{{\mathcal {C}}^\varepsilon }V+\int _{\partial B(0,\rho _\varepsilon )}V. \end{aligned}$$

By taking $V\equiv 1$, one sees that the necessary and sufficient condition for existence of a solution (see for example [40, Theorem 5.7.7]) is

$$\begin{aligned} c_\varepsilon =\frac{A_d \rho _\varepsilon ^{d-1}}{|{\mathcal {C}}^\varepsilon |}\sim A_d (\rho _\varepsilon )^{d-1}. \end{aligned}$$

Uniqueness of the solution is guaranteed by requiring that $\psi _\varepsilon$ be orthogonal to constants on ${\mathcal {C}}^\varepsilon$. Therefore, $\psi _\varepsilon$ is the unique function such that

$$\begin{aligned} {\left\{ \begin{array}{ll} \Delta \psi _\varepsilon =c_\varepsilon &{} \text{ in } {\mathcal {C}}^\varepsilon \\ \partial _\nu \psi _\varepsilon =1&{} \text{ on } \partial B(0,\rho _\varepsilon ) \end{array}\right. } \qquad \text{ and } \qquad \int _{{\mathcal {C}}^\varepsilon }\psi _\varepsilon =0. \end{aligned}$$

Consider the union of all cells strictly contained in $\Omega$,

$$\begin{aligned} {\widetilde{\Omega }}^\varepsilon :=\bigcup _{{\mathbf {k}}\in I^\varepsilon }Q_{{\mathbf {k}}}^\varepsilon \subset \Omega ^\varepsilon . \end{aligned}$$

Define the function $\Psi _\varepsilon : {\mathbb {R}}^d {\setminus } \bigcup _{{\mathbf {k}}\in {\mathbb {Z}}^d} T_{{\mathbf {k}}}^\varepsilon \rightarrow {\mathbb {R}}$ as the scaled lift of $\psi _\varepsilon$. That is, if $q : {\mathbb {R}}^d \rightarrow {\mathbb {R}}^d/{\mathbb {Z}}^d$ is the covering map, then

$$\begin{aligned} \Psi _\varepsilon (\varepsilon {\mathbf {x}}):= \psi _\varepsilon (q({\mathbf {x}})). \end{aligned}$$

This function satisfies

$$\begin{aligned} {\left\{ \begin{array}{ll} \Delta \Psi _\varepsilon = \varepsilon ^{-2} c_\varepsilon &{} \text{ in } {\widetilde{\Omega }}^\varepsilon ,\\ \partial _\nu \Psi _\varepsilon = \varepsilon ^{-1} &{} \text{ on } \partial T^\varepsilon . \end{array}\right. } \end{aligned}$$

#### Lemma 24

The functional $L_\varepsilon :\mathrm {H}^1(\Omega )\rightarrow {\mathbb {R}}$ admits the following representation:

$$\begin{aligned}L_\varepsilon (V)= \varepsilon \int _{{\widetilde{\Omega }}^\varepsilon }\nabla \Psi _\varepsilon \cdot \nabla V \, \mathrm {d}{\mathbf {x}}+ \varepsilon \int _{\partial {\widetilde{\Omega }}^\varepsilon {\setminus } \partial T^\varepsilon } V \partial _\nu \Psi _\varepsilon \, \mathrm {d}A +\varepsilon ^{-1}c_\varepsilon \int _{{\widetilde{\Omega }}^\varepsilon }V \, \mathrm {d}A.\end{aligned}$$

The proof is immediately apparent, since for $V\in \mathrm {H}^1(\Omega )$, the following holds:

107 $$\begin{aligned} \int _{{\widetilde{\Omega }}^\varepsilon }\nabla \Psi _\varepsilon \cdot \nabla V \, \mathrm {d}{\mathbf {x}}= -\varepsilon ^{-2}c_\varepsilon \int _{{\widetilde{\Omega }}^\varepsilon }V \, \mathrm {d}{\mathbf {x}}+\varepsilon ^{-1}\int _{\partial T^\varepsilon }V \, \mathrm {d}A + \int _{\partial {\widetilde{\Omega }}^\varepsilon {\setminus } \partial T^\varepsilon } V \partial _\nu \Psi _\varepsilon \, \mathrm {d}A.\nonumber \\ \end{aligned}$$

We establish the following claim concerning $\psi _\varepsilon$:

#### Lemma 25

There is a constant *C*, depending only on the dimension and on $\beta$, such that

108 $$\begin{aligned} \left\| \psi _\varepsilon \right\| _{\mathrm {H}^1({\mathcal {C}}^\varepsilon )} \leqq C \varepsilon ^{\frac{1}{2} + \frac{1}{d}}. \end{aligned}$$

Furthermore, for any $s > 1$, any compact set $K \subset {\mathcal {C}}$, containing the origin in its interior, there is a constant $C'$ depending only on the dimension, $\beta$, *s*, and on *K* such that

109 $$\begin{aligned} \left\| \psi _\varepsilon \right\| _{\mathrm {H}^s({\mathcal {C}}^\varepsilon {\setminus } K)} \leqq C' \varepsilon ^{\frac{1}{2} + \frac{1}{d}}. \end{aligned}$$

In particular, this implies $\left\| D^\alpha \psi _\varepsilon \right\| _{\mathrm {L}^\infty ({\mathcal {C}}^\varepsilon {\setminus } K)}$ decays as $\varepsilon ^{\frac{1}{2} + \frac{1}{d}}$ for any multi-index $\alpha$.

#### Proof

Observe that since $\psi _\varepsilon$ has mean 0 on ${\mathcal {C}}^\varepsilon$, the Poincaré–Wirtinger inequality implies

110 $$\begin{aligned} \mu _1({\mathcal {C}}^\varepsilon ) \left\| \psi _\varepsilon \right\| ^2_{\mathrm {L}^2({\mathcal {C}}^\varepsilon )} \leqq \left\| \nabla \psi _\varepsilon \right\| ^2_{\mathrm {L}^2({\mathcal {C}}^\varepsilon )^d}, \end{aligned}$$

where $\mu _1({\mathcal {C}}^\varepsilon )$ is the first non-zero Neumann eigenvalue of ${\mathcal {C}}^\varepsilon$. Observe that

$$\begin{aligned} \mu _1({\mathcal {C}}^\varepsilon ) \rightarrow 4\pi ^2\qquad \text {as }\varepsilon \rightarrow 0. \end{aligned}$$

Indeed, $\mu _1({\mathcal {C}}^\varepsilon )$ is the first non-zero Neumann eigenvalue of a punctured *d*-dimensional torus, which is known to converge to the first nonzero eigenvalue of the torus itself as $\varepsilon \searrow 0$; see [6, Chapter IX], for instance.

Take $V = \psi _\varepsilon$ in the variational characterisation (108) of $\psi _\varepsilon$, and consider $\varepsilon$ to be small enough that $\mu _1({\mathcal {C}}^\varepsilon ) \geqq 1$. Using the Cauchy–Schwarz inequality yields

111 $$\begin{aligned} \begin{aligned} \left\| \psi _\varepsilon \right\| _{\mathrm {H}^1({\mathcal {C}}^\varepsilon )}^2&\leqq 2\int _{{\mathcal {C}}^\varepsilon }|\nabla \psi _\varepsilon |^2\, \mathrm {d}{\mathbf {x}}\\&\leqq 2\int _{\partial B(0,\rho _\varepsilon )}\psi _\varepsilon \, \mathrm {d}A\\&\leqq 2\sqrt{A_d \rho _\varepsilon ^{d-1}}\Vert \psi _\varepsilon \Vert _{\mathrm {L}^2(\partial B(0,\rho _\varepsilon ))}\\&\leqq 2\sqrt{A_d\rho _\varepsilon ^{d-1}}\Vert \tau _\varepsilon \Vert \Vert \psi _\varepsilon \Vert _{\mathrm {H}^1({\mathcal {C}}^\varepsilon )}, \end{aligned} \end{aligned}$$

where $\tau _\varepsilon$ is the trace operator $\mathrm {H}^1({\mathcal {C}}^\varepsilon ) \rightarrow \mathrm {L}^2(\partial B(0,\rho _\varepsilon ))$. From the definition of $\tau _\varepsilon$ and monotonicity of the involved integrals, we have that

112 $$\begin{aligned} \left\| \tau _\varepsilon \right\| \leqq \gamma \left( \varepsilon ^{\frac{1}{d-1}},1\right) ^{-1}, \end{aligned}$$

where $\gamma$ is defined in Lemma 13. We therefore deduce that $\left\| \tau _\varepsilon \right\| \ll \varepsilon ^{1/d}$, the implicit constant depending only on the dimension and on $\beta$. Finally, dividing both sides in (113) by $\left\| \psi _\varepsilon \right\| _{\mathrm {H}^1}$, observing that $\rho _\varepsilon ^{d-1} \sim \beta \varepsilon$ and inserting the bound for $\left\| \tau _\varepsilon \right\|$ in (113) finishes the proof of the first inequality.

For the second one, we have from [20, Theorem 8.10] that there is a constant ${{\widetilde{C}}}$ depending only on the dimension, on *K*, and on *s* such that

113 $$\begin{aligned} \left\| \psi _\varepsilon \right\| _{\mathrm {H}^s({\mathcal {C}}^\varepsilon {\setminus } K)} \leqq {{\widetilde{C}}} \left( \left\| \psi _\varepsilon \right\| _{\mathrm {H}^1({\mathcal {C}}^\varepsilon )} + \left\| c_\varepsilon \right\| _{\mathrm {H}^s({\mathcal {C}}^\varepsilon )} \right) . \end{aligned}$$

The second term is of order $\rho _\varepsilon ^{d-1} \sim \varepsilon$, and bounds for the $\mathrm {H}^1$ norm of $\psi _\varepsilon$ that were obtained in (113) conclude the proof of the second inequality.

As for the remark considering the $\mathrm {L}^\infty$ bounds on derivatives of $\psi _\varepsilon$, we have from inequality (111) that

114 $$\begin{aligned} \left\| D^\alpha \psi _\varepsilon \right\| _{\mathrm {H}^{s-\left|\alpha \right|}({\mathcal {C}}^\varepsilon {\setminus } K)} \leqq \left\| \psi _\varepsilon \right\| _{\mathrm {H}^s({\mathcal {C}}^\varepsilon {\setminus } K)} \leqq C'\varepsilon ^{\frac{1}{2} + \frac{1}{d}}. \end{aligned}$$

Choosing $s > \left|\alpha \right|+ \frac{d+1}{2}$, and $\varepsilon$ small enough that ${\mathcal {C}}^\varepsilon {\setminus } K = {\mathcal {C}}{\setminus } K$, we have that

$$\begin{aligned}\mathrm {H}^{s- \left|\alpha \right|}({\mathcal {C}}^\varepsilon {\setminus } K) \hookrightarrow \mathrm {L}^\infty ({\mathcal {C}}^\varepsilon {\setminus } K)\end{aligned}$$

with norm independent of $\varepsilon$, concluding the proof. $\square$

**Step 3**: Computing the limit problem. It follows from Lemma 23 that it is sufficient to verify convergence for a test function $V \in C^\infty ({\overline{\Omega }})$. From the outer representation for $L_\varepsilon$ we have that

115 $$\begin{aligned} \begin{aligned} {{\widetilde{L}}}_\varepsilon (V)&= \int _{\partial T^\varepsilon }u_k^{\varepsilon }V \\&= \varepsilon \int _{{\widetilde{\Omega }}^\varepsilon }\nabla \Psi _\varepsilon \cdot \nabla (u_k^{\varepsilon }V) + \int _{\partial {\widetilde{\Omega }}^\varepsilon {\setminus } \partial T^\varepsilon } u_k^\varepsilon V \partial _\nu \Psi _\varepsilon +\underbrace{\varepsilon ^{-1}c_\varepsilon \int _{{\widetilde{\Omega }}^\varepsilon }u_k^{\varepsilon }V}_{\rightarrow A_d\beta \int _\Omega UV}, \end{aligned}\nonumber \\ \end{aligned}$$

where convergence of the last term stems from strong $\mathrm {L}^2$ convergence. We now show that the two other terms converge to 0. For the first one, the Cauchy–Schwarz inequality gives

116 $$\begin{aligned} \int _{{\widetilde{\Omega }}^\varepsilon } \varepsilon \nabla \Psi _\varepsilon \cdot \nabla (u_k^{\varepsilon }V)\leqq \left( \int _{{\widetilde{\Omega }}^\varepsilon } \varepsilon ^{2}|\nabla \Psi _\varepsilon |^2 \int _{{\widetilde{\Omega }}^\varepsilon }|\nabla (u_k^{\varepsilon }V)|^2\right) ^{1/2}. \end{aligned}$$

Let us first observe that since *V* is in $C^\infty ({\overline{\Omega }})$, we have that

117 $$\begin{aligned} \begin{aligned} \int _{{\widetilde{\Omega }}^\varepsilon } \left|\nabla (u_k^{\varepsilon } V) \right|^2&\leqq 2 \sup _{{\mathbf {x}}\in {\widetilde{\Omega }}^\varepsilon } \left( \left|V({\mathbf {x}}) \right|^2 + \left|\nabla V({\mathbf {x}}) \right|^2 \right) \Vert U_k^{\varepsilon }\Vert _{\mathrm {H}_1(\Omega )} \\&= O_{}\left( 1 \right) . \end{aligned} \end{aligned}$$

Regarding the other term, note that

118 $$\begin{aligned} \begin{aligned} \int _{{\widetilde{\Omega }}^\varepsilon } \varepsilon ^2 |\nabla \Psi _\varepsilon |^2&= \varepsilon ^2 \sum _{{\mathbf {k}}\in I^\varepsilon }\int _{Q^\varepsilon _{{\mathbf {k}}}} |\nabla \Psi _\varepsilon |^2 \\&=\sum _{{\mathbf {k}}\in I^\varepsilon }\varepsilon ^{d}\int _{{\mathcal {C}}^\varepsilon }|\nabla \psi _\varepsilon |^2 \\&\sim |\Omega |\int _{{\mathcal {C}}^\varepsilon }|\nabla \psi _\varepsilon |^2. \end{aligned} \end{aligned}$$

It follows from Lemma 25 that

119 $$\begin{aligned} \int _{{\mathcal {C}}^\varepsilon } \left|\nabla \psi _\varepsilon \right|^2 \, \mathrm {d}{\mathbf {x}}= O_{}\left( \varepsilon ^{1 + \frac{2}{d}} \right) , \end{aligned}$$

hence that term indeed goes to 0. For the second term in (117), we have from the generalised Hölder inequality that

120 $$\begin{aligned} \varepsilon \int _{\partial {\widetilde{\Omega }}^\varepsilon {\setminus } \partial T^\varepsilon } u_k^\varepsilon V \partial _\nu \Psi _\varepsilon \, \mathrm {d}A \leqq \varepsilon \left\| V \right\| _{\mathrm {L}^2(\partial {\widetilde{\Omega }}^\varepsilon {\setminus } \partial T^\varepsilon )} \left\| u_k^\varepsilon \right\| _{\mathrm {L}^2(\partial {\widetilde{\Omega }}^\varepsilon {\setminus } \partial T^\varepsilon )} \left\| \partial _\nu \Psi _\varepsilon \right\| _{\mathrm {L}^\infty (\partial {\widetilde{\Omega }}^\varepsilon {\setminus } \partial T^\varepsilon )}.\nonumber \\ \end{aligned}$$

We now analyse each of those norms.

First, by scaling of derivatives, we have

121 $$\begin{aligned} \begin{aligned} \left\| \varepsilon \partial _\nu \Psi _\varepsilon \right\| _{\mathrm {L}^\infty (\partial \Omega ^\varepsilon {\setminus } \partial T^\varepsilon )}&\leqq \sup _{\left|\alpha \right|= 1}\left\| D^\alpha \psi _\varepsilon \right\| _{\mathrm {L}^\infty ({\mathcal {C}}^\varepsilon {\setminus } B(0,1/4))} \\&\ll \varepsilon ^{\frac{1}{2} + \frac{1}{d}}, \end{aligned} \end{aligned}$$

where the last bound holds by Lemma 25. We move on to $\left\| u_k^\varepsilon \right\| _{\mathrm {L}^2(\partial {\widetilde{\Omega }}^\varepsilon {\setminus } \partial T^\varepsilon )}$. Denote by ${{\widetilde{I}}}^\varepsilon$ the set of indices ${\mathbf {n}}\in {\mathbb {Z}}^d$ such that $\partial Q_{\mathbf {n}}^\varepsilon \cap \partial {\widetilde{\Omega }}^\varepsilon \ne \varnothing$. One can see that

122 $$\begin{aligned} \left\| u_k^\varepsilon \right\| _{\mathrm {L}^2(\partial {\widetilde{\Omega }}^\varepsilon {\setminus } \partial T^\varepsilon )} \leqq \sum _{{\mathbf {n}}\in {{\widetilde{I}}}^\varepsilon }\left\| U_k^\varepsilon \right\| _{\mathrm {L}^2(\partial Q_{\mathbf {n}}^\varepsilon )}. \end{aligned}$$

On the other hand, it follows from Lemma 15 that there is a constant *C* (in fact, the trace constant of the unit cube) such that

123 $$\begin{aligned} \begin{aligned} \sum _{{\mathbf {n}}\in {{\widetilde{I}}}^\varepsilon }\left\| U_k^\varepsilon \right\| _{\mathrm {L}^2(\partial Q_{\mathbf {n}}^\varepsilon )}&\leqq C\varepsilon ^{-1/2} \sum _{{\mathbf {n}}\in {{\widetilde{I}}}^\varepsilon }\left\| U_k^\varepsilon \right\| _{\mathrm {H}^1(Q_{{\mathbf {n}}}^\varepsilon )} \\&\leqq C\varepsilon ^{-1/2} \left\| U_k^\varepsilon \right\| _{\mathrm {H}^1(\Omega )}. \end{aligned} \end{aligned}$$

Finally, since *V* is fixed and smooth, we have that

124 $$\begin{aligned} \begin{aligned} \left\| V \right\| _{\mathrm {L}^2(\partial {\widetilde{\Omega }}^\varepsilon {\setminus } \partial T^\varepsilon )}&\leqq \left|\partial {\widetilde{\Omega }}^\varepsilon {\setminus } \partial T^\varepsilon \right|^{1/2} \sup _{{\mathbf {x}}} V({\mathbf {x}}) \\&\leqq C \left|\partial \Omega \right|^{1/2} \sup _{\mathbf {x}}V({\mathbf {x}}). \end{aligned} \end{aligned}$$

All in all, this implies that the second term in (117) is bounded by a constant times $\varepsilon ^{1/d}$, hence goes to 0 as $\varepsilon \rightarrow 0$, finishing the proof of Lemma 20. $\square$

We are now ready to complete the proof of the main result of this subsection.

#### Proof of Proposition 19

We know from [42] that we only need to show that the solutions converge to a solution $(\Sigma ,U)$ of the weak formulation of Problem 4,1 which is that for any test function *V*,

125 $$\begin{aligned} \int _\Omega \nabla U \cdot \nabla V \, \mathrm {d}{\mathbf {x}}= \Sigma \left( A_d \beta \int _\Omega U V \, \mathrm {d}{\mathbf {x}}+ \int _{\partial \Omega } U V \, \mathrm {d}A\right) . \end{aligned}$$

The weak formulation of the Steklov problem on $\Omega ^\varepsilon$ is that for all test functions *V*,

126 $$\begin{aligned} \int _{\Omega ^\varepsilon } \nabla u_k^\varepsilon \cdot \nabla V \, \mathrm {d}{\mathbf {x}}= \sigma _k^\varepsilon \left( \int _{\partial \Omega } u_k^\varepsilon V \, \mathrm {d}A + \int _{\partial T^\varepsilon } u_k^\varepsilon V \, \mathrm {d}A\right) . \end{aligned}$$

The convergence of the gradient terms follows from weak convergence in $\mathrm {H}^1$ of $U_k^\varepsilon$ to *U* and Lemma 12. We already have that $\sigma _k^\varepsilon \rightarrow \Sigma$. The integrals on $\partial \Omega$ converge by weak convergence in $\mathrm {H}^1$ and compactness of the trace operator on $\partial \Omega$. Finally, convergence of the interior term comes from Proposition 20. $\square$

### Spectral convergence of the problem

We need the following technical lemma:

#### Lemma 26

As $\varepsilon \rightarrow 0$, we have that

127 $$\begin{aligned} {{\widetilde{L}}}_\varepsilon (u_k^\varepsilon ) \rightarrow A_d \beta \int _{\Omega } U^2 \, \mathrm {d}{\mathbf {x}}. \end{aligned}$$

#### Remark 27

Observe that this situation is specific to the sequence $u_k^\varepsilon$. Indeed, there are sequences $\left\{ v_\varepsilon \right\}$ converging weakly to some $v \in \mathrm {H}^1(\Omega )$ such that

128 $$\begin{aligned} \lim _{\varepsilon \rightarrow 0} {{\widetilde{L}}}_\varepsilon (v_\varepsilon ) \ne A_d \beta \int _\Omega U v \, \mathrm {d}{\mathbf {x}}. \end{aligned}$$

#### Proof

From the outer representation (117), we have that

129 $$\begin{aligned} \begin{aligned} {{\widetilde{L}}}_\varepsilon (u_k^\varepsilon )&= \varepsilon \int _{{\widetilde{\Omega }}^\varepsilon }\nabla \Psi _\varepsilon \cdot \nabla (u_k^{\varepsilon })^2 \, \mathrm {d}{\mathbf {x}}+ \varepsilon \int _{\partial {\widetilde{\Omega }}^\varepsilon {\setminus } \partial T^\varepsilon } (u_k^\varepsilon )^2 \partial _\nu \Psi _\varepsilon \, \mathrm {d}{\mathbf {x}}+ \varepsilon ^{-1}c_\varepsilon \int _{{\widetilde{\Omega }}^\varepsilon }(u_k^{\varepsilon })^2 \, \mathrm {d}{\mathbf {x}}, \\&= \varepsilon \int _{{\widetilde{\Omega }}^\varepsilon }2\nabla \Psi _\varepsilon \cdot u_k^\varepsilon \nabla u_k^\varepsilon \, \mathrm {d}{\mathbf {x}}+\varepsilon \int _{\partial {\widetilde{\Omega }}^\varepsilon {\setminus } \partial T^\varepsilon } (u_k^\varepsilon )^2 \partial _\nu \Psi _\varepsilon \, \mathrm {d}{\mathbf {x}}+\varepsilon ^{-1}c_\varepsilon \int _{{\widetilde{\Omega }}^\varepsilon }(u_k^{\varepsilon })^2 \, \mathrm {d}{\mathbf {x}}. \\ \end{aligned} \end{aligned}$$

The last term converges towards the desired $A_d \beta \int _\Omega U^2 \, \mathrm {d}{\mathbf {x}}$ once again by strong $L^2$ convergence of the sequence $U_k^\varepsilon$. To study the first term, let us now introduce the sets

130 $$\begin{aligned} \omega ^\varepsilon := \bigcup _{{\mathbf {k}}\in I^\varepsilon } B\left( \varepsilon {\mathbf {k}},\frac{\varepsilon }{4}\right) {\setminus } B(\varepsilon {\mathbf {k}},r_\varepsilon ) \subset {\widetilde{\Omega }}^\varepsilon , \end{aligned}$$

and decompose

131 $$\begin{aligned} \varepsilon \int _{{\widetilde{\Omega }}^\varepsilon } \nabla \Psi _\varepsilon \cdot \nabla (u_k^\varepsilon )^2 \, \mathrm {d}{\mathbf {x}}= 2 \left( \int _{\omega ^\varepsilon } + \int _{{\widetilde{\Omega }}^\varepsilon {\setminus } \omega ^\varepsilon } \right) \varepsilon \nabla \Psi _\varepsilon \cdot u_k^\varepsilon \nabla u_k^\varepsilon \, \mathrm {d}{\mathbf {x}}. \end{aligned}$$

Let us first consider the integral over $\omega ^\varepsilon$. It follows from [5] that the $\mathrm {L}^\infty$ norms of the Steklov eigenfunctions $u_k^\varepsilon$ is bounded, uniformly in terms of $\sigma _k^\varepsilon$, and the norm of the trace $T : \mathrm {B}\mathrm {V}(\Omega ^\varepsilon ) \rightarrow \mathrm {L}^1(\partial \Omega ^\varepsilon )$, which we have shown in Lemma 16 to be bounded. This, along with the scaled $\mathrm {H}^1$ norm estimate for $\varepsilon \nabla \Psi _\varepsilon$ from Lemma 25, the $\mathrm {L}^2$ boundedness of $\nabla u_k^\varepsilon$ from Lemma 18 and the generalised Hölder inequality yields

132 $$\begin{aligned} \begin{aligned} \int _{\omega ^\varepsilon }2 \varepsilon \nabla \Psi _\varepsilon \cdot u_k^\varepsilon \nabla u_k^\varepsilon \, \mathrm {d}{\mathbf {x}}&\leqq 2 \left\| \varepsilon \nabla \Psi _\varepsilon \right\| _{\mathrm {L}^2(\Omega ^\varepsilon )^d} \left\| \nabla u_k^\varepsilon \right\| _{\mathrm {L}^2(\Omega ^\varepsilon )^d} \left\| u_k^\varepsilon \right\| _{\mathrm {L}^\infty (\omega ^\varepsilon )} \\&\ll \varepsilon ^{\frac{1}{2} + \frac{1}{d}}. \end{aligned} \end{aligned}$$

For the integral over ${\widetilde{\Omega }}^\varepsilon {\setminus } \omega ^\varepsilon$, observe that scaling Lemma 25 yields

133 $$\begin{aligned} \begin{aligned} \left\| \varepsilon \nabla \Psi _\varepsilon \right\| _{\mathrm {L}^\infty ({\widetilde{\Omega }}^\varepsilon {\setminus } \omega ^\varepsilon )^d}&= \sup _{\left|\alpha \right|= 1} \left\| D^\alpha \psi _\varepsilon \right\| _{\mathrm {L}^\infty ({\mathcal {C}}^\varepsilon {\setminus } B(0,1/4))} \\&\ll \varepsilon ^{\frac{1}{2} + \frac{1}{d}}. \end{aligned} \end{aligned}$$

Inserting that bound into the generalised Hölder inequality, along with $\mathrm {H}^1$ boundedness of $u_k^\varepsilon$ yields

134 $$\begin{aligned} \begin{aligned} \int _{\omega ^\varepsilon }2 \varepsilon \nabla \Psi _\varepsilon \cdot u_k^\varepsilon \nabla u_k^\varepsilon \, \mathrm {d}{\mathbf {x}}&\leqq 2 \left\| \varepsilon \nabla \Psi _\varepsilon \right\| _{\mathrm {L}^\infty ({\widetilde{\Omega }}^\varepsilon {\setminus } \omega ^\varepsilon )^d} \left\| \nabla u_k^\varepsilon \right\| _{\mathrm {L}^2(\Omega ^\varepsilon )^d} \left\| u_k^\varepsilon \right\| _{\mathrm {L}^2(\Omega ^\varepsilon )} \\&\ll \varepsilon ^{\frac{1}{2} + \frac{1}{d}}. \end{aligned} \end{aligned}$$

Finally, for the integral on the boundary $\partial {\widetilde{\Omega }}^\varepsilon {\setminus } \partial T^\varepsilon$, we have from Hölder that

135 $$\begin{aligned} \varepsilon \int _{\partial {\widetilde{\Omega }}^\varepsilon {\setminus } \partial T^\varepsilon } (u_k^\varepsilon )^2 \partial _\nu \Psi _\varepsilon \, \mathrm {d}A \leqq \varepsilon \left|\partial {\widetilde{\Omega }}^\varepsilon {\setminus } \partial T^\varepsilon \right|\left\| \partial _\nu \Psi _\varepsilon \right\| _{\mathrm {L}^\infty (\partial {\widetilde{\Omega }}^\varepsilon {\setminus } \partial T^\varepsilon )}\left\| (u_k^\varepsilon )^2 \right\| _{\mathrm {L}^\infty (\partial {\widetilde{\Omega }}^\varepsilon {\setminus } \partial T^\varepsilon )}. \end{aligned}$$

We have as in the proof of Lemma 20 that the $\mathrm {L}^2$ norm of $u_k^\varepsilon$ is uniformly bounded, from uniform boundedness of the trace operator. From Lemma 25 we have that

$$\begin{aligned} \varepsilon \left\| \partial _\nu \Psi _\varepsilon \right\| _{\mathrm {L}^\infty (\partial {\widetilde{\Omega }}^\varepsilon {\setminus } \partial T^\varepsilon )} = O_{}\left( \varepsilon ^{\frac{1}{2} + \frac{1}{d}} \right) , \end{aligned}$$

and as earlier, we have

$$\begin{aligned} \left\| (u_k^\varepsilon )^2 \right\| _{\mathrm {L}^\infty (\partial {\widetilde{\Omega }}^\varepsilon {\setminus } \partial T^\varepsilon )} \leqq C, \end{aligned}$$

from Lemma 16. Finally, it follows from standard lattice packing theory that $\# {{\widetilde{I}}}^\varepsilon \leqq C \varepsilon ^{1-d}$ for some *C* depending only on $\Omega$, so that $\left|\partial {\widetilde{\Omega }}^\varepsilon {\setminus } \partial T^\varepsilon \right| \leqq C$. Combining these three estimates, we have indeed that the product in (137) is going to 0 as $\varepsilon \rightarrow 0$, concluding the proof. $\square$

Until now, we have shown that the harmonic extensions to the holes in $\Omega ^\varepsilon$ of Steklov eigenpairs $(\sigma _k^{\varepsilon },u_k^{\varepsilon })$ converge weakly in $\mathrm {H}^1$ and strongly in $\mathrm {L}^2$ to a solution $(\Sigma ,U)$ to the problem

136 $$\begin{aligned} {\left\{ \begin{array}{ll} - \Delta U = A_d \beta \Sigma U &{} \text {in } \Omega , \\ \partial _\nu U = \Sigma U &{} \text {on } \partial \Omega . \end{array}\right. } \end{aligned}$$

It remains to be shown that the convergence is to the “right” eigenpair $(\Sigma _{k,\beta },U_k)$. Spectral convergence of this type is a staple of homogenisation theory, see for example [2, 37] in a standard setting or [16] using the theory of *E*-convergence. In both cases, the general theory cannot be directly applied here since the Hilbert space $\mathrm {L}^2_{A_d\beta }(\Omega ) \times \mathrm {L}^2(\partial \Omega )$ on which the limit problem is self-adjoint has no natural embedding to the Hilbert spaces $\mathrm {L}^2(\partial \Omega ^\varepsilon )$, compare for example with [16, Definitions 1–3]. Our methods use instead the quadratic forms associated with the eigenproblems directly, similar methods were used in spectral prescription, see for example [13].

For the remainder of this section, as $\beta$ is fixed, we will write simply $\Sigma _k$. We first start by the following lemma, showing that the limit function *U* does not degenerate to the 0 function:

#### Lemma 28

Let *U* be such that $U_k^{\varepsilon } \rightarrow U$ weakly in $\mathrm {H}^1(\Omega )$. Then,

137 $$\begin{aligned} (U,U)_\beta = 1. \end{aligned}$$

#### Proof

By compactness of the trace operator on $\partial \Omega$, we have that

138 $$\begin{aligned} \int _{\partial \Omega } (U_k^{\varepsilon })^2 \, \mathrm {d}{\mathbf {x}}\rightarrow \int _{\partial \Omega } U^2 \, \mathrm {d}{\mathbf {x}}. \end{aligned}$$

as $\varepsilon \rightarrow 0$. Moreover, by Lemma 26, we have that

139 $$\begin{aligned} \int _{\partial T^\varepsilon } \left( U_k^\varepsilon \right) ^2 \, \mathrm {d}{\mathbf {x}}\rightarrow A_d\beta \int _\Omega U^2\, \mathrm {d}{\mathbf {x}}\end{aligned}$$

as $\varepsilon \rightarrow 0$. Hence $(U_k^\varepsilon ,U_k^\varepsilon )_{\partial ^\varepsilon } \rightarrow (U,U)_\beta$. Since $U_k^\varepsilon$ has been normalised to $\mathrm {L}^2(\partial \Omega ^\varepsilon )$ norm 1, this concludes the proof. $\square$

We are now ready to complete the proof of our first main result.

#### Proof of Theorem 2

We first show that all the eigenvalues converge. We proceed by induction on the eigenvalue rank *k*. The case $k = 0$ is trivial. Indeed, we then have that the eigenvalues $\sigma _0^{\varepsilon } \equiv 0$ obviously converge to $\Sigma _0 = 0$ and the normalised eigenfunctions $U_0^{\varepsilon }({\mathbf {x}}) = \left|\partial \Omega ^\varepsilon \right|^{-1/2}$, which converges to the constant fonction

$$\begin{aligned} U_0({\mathbf {x}}) = \left( \left|\partial \Omega \right| + A_d\beta \left|\Omega \right|\right) ^{-1/2}. \end{aligned}$$

Suppose now that for all $0 \leqq j \leqq k-1$, we have that $U_j^{\varepsilon }$ converges to $U_j$ weakly in $\mathrm {H}^1(\Omega )$. We first show that

140 $$\begin{aligned} \Sigma _k \geqq \sigma _k^{\varepsilon } + o_{}\left( 1 \right) . \end{aligned}$$

In order to do this, we will show that the eigenfunctions $U_k$ are good approximations to appropriate test functions for the variational characterisation (18) of $\sigma _k^\varepsilon$.

Observe that by compactness of the trace operator on $\partial \Omega$ and by Lemma 20,

141 $$\begin{aligned} \lim _{\varepsilon \rightarrow 0} \int _{\partial \Omega ^\varepsilon } u_j^{\varepsilon } U_k \, \mathrm {d}A = \int _{\partial \Omega } U_k U_j\, \mathrm {d}A + A_d\beta \int _\Omega U_k U_j \, \mathrm {d}{\mathbf {x}}= 0 \end{aligned}$$

for all $0 \leqq j \leqq k-1$. Hence, we can write

142 $$\begin{aligned} U_k = V^{\varepsilon } + \sum _{j=0}^{k-1} \eta _j^{\varepsilon } u_j^{\varepsilon } \, \mathrm {d}A, \end{aligned}$$

where for all $0 \leqq j \leqq k-1$ and all $\varepsilon > 0$,

143 $$\begin{aligned} \int _{\partial \Omega ^\varepsilon } V^\varepsilon u_j^\varepsilon = 0, \end{aligned}$$

and $\eta _j^{\varepsilon } \rightarrow 0$ as $\varepsilon \rightarrow 0$. Now, we have that

144 $$\begin{aligned} \begin{aligned} \Sigma _k&= \int _\Omega \left|\nabla U_k \right|^2 \, \mathrm {d}{\mathbf {x}}\\&\geqq \int _{\Omega ^\varepsilon } \left|\nabla V^{\varepsilon } \right|^2 + \sum _{j=0}^{k-1}(\eta _j^{\varepsilon }) \nabla u_j^{\varepsilon } \cdot \nabla V^\varepsilon + \sum _{j,l = 0}^{k-1} \eta _j^\varepsilon \eta _k^\varepsilon \nabla u_j^\varepsilon \cdot \nabla u_k^\varepsilon \, \mathrm {d}{\mathbf {x}}. \\ \end{aligned} \end{aligned}$$

Since the $u_j^\varepsilon$ and $V^\varepsilon$ are bounded in $\mathrm {H}^1(\Omega )$, the integral of the two sums in the previous equation go to 0 as $\varepsilon \rightarrow 0$. On the other hand, we have that for all $\varepsilon > 0$, $V^\varepsilon$ is an appropriate test function for $\sigma _k^\varepsilon$. Hence,

145 $$\begin{aligned} \int _{\Omega ^\varepsilon } \left|\nabla V^\varepsilon \right|^2 \, \mathrm {d}{\mathbf {x}}\geqq \sigma _k^\varepsilon \int _{\partial \Omega ^\varepsilon } (V^\varepsilon )^2 \, \mathrm {d}A. \end{aligned}$$

It follows from the decomposition (144) and the fact that, by Lemma 20,

146 $$\begin{aligned} \int _{\partial \Omega ^\varepsilon } U_k^2 \, \mathrm {d}A \rightarrow \int _{\partial \Omega } U_k^2 \, \mathrm {d}A + A_d\beta \int _\Omega U_k^2 \, \mathrm {d}{\mathbf {x}}= 1 \end{aligned}$$

that

147 $$\begin{aligned} \lim _{\varepsilon \rightarrow 0} \int _{\partial \Omega ^\varepsilon } (V^\varepsilon )^2 \, \mathrm {d}{\mathbf {x}}= 1, \end{aligned}$$

implying that indeed $\Sigma _k \geqq \sigma _k^{\varepsilon } + o_{}\left( 1 \right)$. We now show that

$$\begin{aligned} \Sigma _k \leqq \sigma _k^{\varepsilon }. \end{aligned}$$

Let $(\Sigma ,U)$ be the limit eigenpair for $(\sigma _k^{\varepsilon },u_k^{\varepsilon })$ and suppose that

$$\begin{aligned} (\Sigma ,U) = (\Sigma _j,U_j) \end{aligned}$$

for some $0 \leqq j \leqq k-1$. We have that

148 $$\begin{aligned} \begin{aligned} 0&= \lim _{\varepsilon \rightarrow 0} \int _{\partial \Omega ^\varepsilon } u_k^{\varepsilon } \, \mathrm {d}A\\&= \lim _{\varepsilon \rightarrow 0}\int _{\partial \Omega ^\varepsilon } u_k^\varepsilon U_j \, \mathrm {d}A + \int _{\partial \Omega ^\varepsilon } u_k^\varepsilon ( u_j^\varepsilon - U_j) \, \mathrm {d}A \end{aligned} \end{aligned}$$

The first term converges to 1 by the assumption that $(U,U_j)_\beta = 1$ and Lemma 20. As for the second term, we have by the Cauchy–Schwarz inequality and the normalisation $\left\| u_k^\varepsilon \right\| _{\mathrm {L}^2(\partial \Omega ^\varepsilon )}=1$ that

149 $$\begin{aligned} \begin{aligned} \int _{\partial \Omega ^\varepsilon } u_k^\varepsilon ( u_j^\varepsilon - U_j) \, \mathrm {d}A&\leqq \left\| u_j^\varepsilon - U_j \right\| _{\mathrm {L}^2(\partial \Omega ^\varepsilon )} \\&\rightarrow 0 \end{aligned} \end{aligned}$$

as $\varepsilon \rightarrow 0$ by Lemma 26. This results in a contradiction. We therefore deduce that $\sigma _k^{\varepsilon }$ converges to some eigenvalue $\Sigma$ of problem 4 that is larger than $\Sigma _j$ for all $j \leqq k-1$. Combining this with the bound (142) implies that $\sigma _k^{\varepsilon }$ converges to $\Sigma _k$, and weak convergence of the eigenfunction therefore follows, when the eigenvalues are simple.

To obtain strong convergence of the eigenfunctions, it is only left to prove that the Dirichlet energy converges. This follows directly from Lemma 12 which tells us, as in (92), that

$$\begin{aligned} {\mathcal {D}}(U_k^\varepsilon ;\Omega ) = {\mathcal {D}}(u_k^\varepsilon ;\Omega ^\varepsilon )(1 + o_{}\left( 1 \right) ), \end{aligned}$$

as well as the fact that

150 $$\begin{aligned} {\mathcal {D}}(u_k^\varepsilon ;\Omega ^\varepsilon ) = \sigma _k^\varepsilon \rightarrow \Sigma _k = {\mathcal {D}}(U_k;\Omega ). \end{aligned}$$

If $\Sigma _k$ has multiplicity *m*, *that is*

151 $$\begin{aligned} \Sigma _{k-1}< \Sigma _k = \cdots = \Sigma _{k+m-1} < \Sigma _{k+m}, \end{aligned}$$

observe that the above argument still yields convergence of $\sigma _j^\varepsilon$ to $\Sigma _j$ for all $k \leqq j < k+m$. For the eigenfunctions, start by fixing a basis $U_k,\dotsc ,U_{k+m - 1}$ of the eigenspace associated with $\Sigma _k$. Observe that along any subsequence there is a further subsequence such that all the eigenfunctions $U_j^\varepsilon$, converge simultaneously to solutions of Problem 4. Since, for all $\varepsilon$, the functions $U_j^\varepsilon$ were $\mathrm {L}^2(\partial \Omega ^\varepsilon )$-orthogonal, in the limit they are still orthogonal, this time with respect to $(\cdot ,\cdot )_\beta$. This implies that in the limit they span the eigenspace associated with $\Sigma _k$. As such, the projection on the span of $\left\{ U_j^\varepsilon : k \leqq j < k+m \right\}$ converges to the projections on the span of $\left\{ U_j : k \leqq j < k+m \right\}$. Since this was true along any subsequence, it is also true for the whole sequence, proving convergence of the projections in the sense alluded to in Remark 3. $\square$

## Dynamical boundary conditions with large parameter

The goal of this final section is to understand the limit, as $\beta$ becomes large, of the eigenvalues $\Sigma _{k,\beta }$, and of the corresponding eigenfunctions $U_{k,\beta }$, normalized by

$$\begin{aligned} 1 = (U_{k,\beta },U_{k,\beta })_\beta = \int _{\partial \Omega } U_{k,\beta }^2 \, \mathrm {d}A + A_d \beta \int _{\Omega } U_{k,\beta }^2 \, \mathrm {d}{\mathbf {x}}. \end{aligned}$$

Recall that the Neumann eigenvalues of $\Omega$ are

$$\begin{aligned} 0=\mu _0\leqq \mu _1\leqq \mu _2\leqq \cdots \nearrow \infty . \end{aligned}$$

We are now ready to prove our second main result.

### Proof of Theorem 5

For *k* fixed, we start by showing that $\beta \Sigma _{k,\beta }$ is bounded. Consider the min–max characterisation of $\Sigma _{k,\beta }$ to obtain

152 $$\begin{aligned} {\widetilde{\Sigma }}_{k,\beta }:=\beta \Sigma _{k,\beta } = \min _{\begin{array}{c} E \subset \mathrm {H}^1(\Omega ) \\ {\text {dim}}(E) = k+1 \end{array}} \max _{f \in E {\setminus } \left\{ 0 \right\} } \frac{\int _{\Omega } \left|\nabla f \right|^2 \, \mathrm {d}{\mathbf {x}}}{\frac{1}{\beta }\int _{\partial \Omega } f^2 \, \mathrm {d}A + A_d \int _\Omega f^2 \, \mathrm {d}{\mathbf {x}}}. \end{aligned}$$

The quotient on the righthand side of (154) is clearly bounded uniformly in $\beta$ for any $k+1$ dimensional subspace of smooth functions on $\Omega$. We can therefore suppose that a subsequence in $\beta$ of ${\widetilde{\Sigma }}_{k,\beta }$ converges, say to ${\widetilde{\Sigma }}_{k,\infty }$. Let us now prove that ${{\widetilde{U}}}_{k,\beta } := \beta ^{1/2} U_{k,\beta }$ is a bounded family in $\mathrm {H}^1(\Omega )$. The normalisation on $U_{k,\beta }$ implies that

$$\begin{aligned} 1 \geqq A_d \beta \int _\Omega U_{k,\beta }^2 \, \mathrm {d}{\mathbf {x}}= A_d \int _\Omega {{\widetilde{U}}}_{k,\beta }^2 \, \mathrm {d}{\mathbf {x}}. \end{aligned}$$

For the Dirichlet energy, we have that

153 $$\begin{aligned} \int _{\Omega } \left|\nabla {{\widetilde{U}}}_{k,\beta } \right|^2 \, \mathrm {d}{\mathbf {x}}= \beta \Sigma _{k,\beta }, \end{aligned}$$

which was already shown to be bounded. Therefore there is also a weakly convergent subsequence in in $\mathrm {H}^1(\Omega )$ as $\beta \rightarrow \infty$, converging to say ${{\widetilde{U}}}_{k,\infty }$. We take the subsequence to coincide with the one for ${\widetilde{\Sigma }}_{k,\beta }$. Observe as well that the normalisation condition on $U_{k,\beta }$ prevents the limit ${{\widetilde{U}}}_{k,\infty }$ from vanishing identically.

The functions ${{\widetilde{U}}}_{k,\beta }$ satisfy the following weak variational characterisation for any element $V \in \mathrm {H}^1(\Omega )$:

154 $$\begin{aligned} \int _{\Omega } \nabla {{\widetilde{U}}}_{k,\beta } \cdot \nabla V\,\, \mathrm {d}{\mathbf {x}}= {\widetilde{\Sigma }}_{k,\beta }\left( \beta ^{-1}\int _{\partial \Omega } {{\widetilde{U}}}_{k,\beta } V \, \mathrm {d}A + A_d \int _\Omega {{\widetilde{U}}}_{k,\beta } V \, \mathrm {d}{\mathbf {x}}\right) . \end{aligned}$$

Letting $\beta \rightarrow \infty$, weak convergence of ${{\widetilde{U}}}_{k,\beta }$ in $\mathrm {H}^1(\Omega )$ implies that the limit satisfies the weak identity

155 $$\begin{aligned} \int _\Omega \nabla {{\widetilde{U}}}_{k,\infty } \cdot \nabla V\,\, \mathrm {d}{\mathbf {x}}= {\widetilde{\Sigma }}_{k,\infty } A_d \int _\Omega {{\widetilde{U}}}_{k,\infty } V \, \mathrm {d}{\mathbf {x}}. \end{aligned}$$

In other words, ${{\widetilde{U}}}_{k,\infty }$ is a solution to the Neumann eigenvalue problem with eigenvalue $\mu = {\widetilde{\Sigma }}_{k,\infty } A_d$.

We now proceed by recursion on *k* to show convergence to the right eigenpair. Once again, the statement is trivial for $k = 0$ and the constant eigenfunction. Assume that we have convergence for the first $k-1$ eigenpairs. We now proceed in a similar fashion as in the proof of the spectral convergence to Problem (4). We repeat the argument because the inequalities are more subtle. We first show that

156 $$\begin{aligned} {\widetilde{\mu }}_k(\Omega ):= \frac{\mu _k(\Omega )}{A_d} \geqq {\widetilde{\Sigma }}_{k,\beta }(1 + o_{}\left( 1 \right) ). \end{aligned}$$

Write

157 $$\begin{aligned} f_k = F_\beta + \sum _{j=0}^{k-1} (f_k, U_{j,\beta })_\beta U_{j,\beta }, \end{aligned}$$

with $F_\beta \perp _\beta U_{j,\beta }$ for $0 \leqq j < k$. We have that

158 $$\begin{aligned} (f_k,U_{j,\beta })_\beta = (f_k,\beta ^{-1/2}f_j)_\beta + (f_k,U_{j,\beta }-\beta ^{-1/2}f_j)_\beta . \end{aligned}$$

The first inner product develops as

159 $$\begin{aligned} (f_k,\beta ^{-1/2}f_j)_\beta = \beta ^{-1/2} \int _{\partial \Omega } f_k f_j \, \mathrm {d}A +A_d\beta ^{1/2}\int _{\Omega } f_k f_j \, \mathrm {d}{\mathbf {x}}. \end{aligned}$$

The first term clearly goes to 0 as $\beta \rightarrow \infty$, and the second one vanishes by orthogonality of the Neumann eigenfunctions in $\mathrm {L}^2(\Omega )$. We now turn our attention to the second inner product in (160). We have that

160 $$\begin{aligned} \lim _{\beta \rightarrow \infty } \beta ^{-1/2} \int _{\partial \Omega }f_k ({{\widetilde{U}}}_{j,\beta } - f_j) \, \mathrm {d}A = 0 \end{aligned}$$

by weak $\mathrm {H}^1$ convergence of ${{\widetilde{U}}}_{j,\beta }$ and compactness of the trace operator. On the other hand, strong $\mathrm {L}^2$ convergence implies that

161 $$\begin{aligned} A_d \beta ^{1/2} \int _{\Omega } f_k ({{\widetilde{U}}}_{j,\beta } - f_j) \, \mathrm {d}{\mathbf {x}}= o_{}\left( \beta ^{1/2} \right) . \end{aligned}$$

All in all, this implies that

162 $$\begin{aligned} (f_k,U_{j,\beta })_\beta = o_{}\left( \beta ^{1/2} \right) \end{aligned}$$

for all $0 \leqq j < k$. We now write

163 $$\begin{aligned} \begin{aligned} {\widetilde{\mu }}_k&= \frac{1}{A_d} \int _\Omega \left|\nabla f_k \right|^2 \, \mathrm {d}{\mathbf {x}}\\&\geqq \frac{1}{A_d} \int _{\Omega } \left|\nabla F_\beta \right|^2 \, \mathrm {d}{\mathbf {x}}- \frac{1}{A_d}\sum _{j=0}^{k-1} (f_k,U_{j,\beta })_\beta ^2 \Sigma _{j,\beta }. \end{aligned} \end{aligned}$$

Since $\beta \Sigma _{j,\beta }$ is bounded and equation (163) implies that $(f_k,U_{j,\beta })_\beta ^2 = o_{}\left( \beta \right)$, we deduce that the last term in (165) goes to 0. We now observe that by the variational characterisation of $\Sigma _{k,\beta }$,

164 $$\begin{aligned} \begin{aligned} \frac{1}{A_d}\int _{\Omega } \left|\nabla F_\beta \right|^2 \, \mathrm {d}{\mathbf {x}}&\geqq \frac{\Sigma _{k,\beta }}{A_d}\left( \int _{\partial \Omega } F_\beta ^2 + A_d \beta \int _\Omega F_\beta ^2\right) , \\&= {\widetilde{\Sigma }}_{k,\beta } \left( \frac{1}{A_d\beta }\int _{\partial \Omega } F_\beta ^2 \, \mathrm {d}A + \int _\Omega F_\beta ^2 \, \mathrm {d}{\mathbf {x}}\right) . \end{aligned} \end{aligned}$$

In the same way as we obtained the bound on the last term in (165), the first integral is $o_{}\left( \beta \right)$. As for the second one, we write

$$\begin{aligned} \begin{aligned} \int _\Omega F_\beta ^2 \, \mathrm {d}{\mathbf {x}}&= \int _{\Omega } \left( f_k - \sum _{j=0}^{k-1} (f_k, U_{j,\beta })_\beta U_{j,\beta }^2\right) ^2 \, \mathrm {d}{\mathbf {x}}\\&= 1 - \sum _{j=0}^{k-1} \int _\Omega (f_k,U_{j,\beta })_\beta f_j U_{j,\beta }\, \mathrm {d}{\mathbf {x}}\\&\quad + \sum _{j,\ell = 0}^{k-1} \int _\Omega (f_k,U_{j,\beta })_\beta (f_k,U_{\ell ,\beta })_\beta U_{j,\beta }U_{\ell ,\beta } \, \mathrm {d}{\mathbf {x}}. \end{aligned} \end{aligned}$$

Strong $\mathrm {L}^2(\Omega )$ convergence of $\beta ^{1/2} U_{j,\beta }$ and the fact that $(f_k,U_{j,\beta }) = o_{}\left( \beta ^{1/2} \right)$ imply that the last two integrals converge to 0 as $\beta \rightarrow \infty$.

We have therefore obtained that

165 $$\begin{aligned} {\widetilde{\mu }}_k(\Omega ) \geqq {\widetilde{\Sigma }}_{k,\beta }\left( 1 + o_{}\left( 1 \right) \right) , \end{aligned}$$

*that is* we have indeed proven assertion (158).

Suppose now that $({\widetilde{\Sigma }}_{k,\beta }, {{\widetilde{U}}}_{k,\beta })$ converge to a Neumann eigenpair $({\widetilde{\mu }}_j, f_j)$ for some $j < k$. Then, we have that

166 $$\begin{aligned} \begin{aligned} 1&= \lim _{\beta \rightarrow \infty } \int _\Omega f_j {{\widetilde{U}}}_{k,\beta } \, \mathrm {d}{\mathbf {x}}\\&= \lim _{\beta \rightarrow \infty } \int _{\Omega } (f_j - {{\widetilde{U}}}_{j,\beta }){{\widetilde{U}}}_{k,\beta } \, \mathrm {d}x + \int _\Omega {{\widetilde{U}}}_{k,\beta } U_{j,\beta } \, \mathrm {d}{\mathbf {x}}\\&\leqq \lim _{\beta \rightarrow \infty }\left\| f_j - {{\widetilde{U}}}_{j,\beta } \right\| _{\mathrm {L}^2(\Omega }\left\| {{\widetilde{U}}}_{k,\beta } \right\| _{\mathrm {L}^2(\Omega )} + \left|\int _\Omega U_{k,\beta } U_{j,\beta } \, \mathrm {d}{\mathbf {x}} \right| \\&= 0, \end{aligned}, \end{aligned}$$

where the limit comes from strong convergence in $\mathrm {L}^2(\partial \Omega )$ to 0 of $U_{j,\beta }$ and our recursion hypothesis. This is a contradiction, hence the convergence is to the correct eigenpair. Strong convergence then follows in the same way as in (152). This implies convergence of the whole sequence if the Neumann spectrum of $\Omega$ is simple. If there are eigenvalues with multiplicity, the same procedure as for the homogenisation problem yields once again convergence.

As for continuity in $\beta$, the same proof goes through in exactly the same way, except for the fact that we do not need to show the boundedness results in $\beta$. $\square$

We can now prove the comparison results between Steklov and Neumann eigenvalues.

### Proof of Corollary 7

It is proved in [11, Theorem 1.4] that any bounded domain $\Omega \subset {\mathbb {R}}^d$ satisfies

167 $$\begin{aligned} \sigma _k(\Omega )|\partial \Omega |\leqq C(d)|\Omega |^{\frac{d-2}{d}}k^{2/d}, \end{aligned}$$

where *C*(*d*) is a constant which depends only on the dimension. When applied to $\Omega ^\varepsilon$ this leads, after taking $\varepsilon \rightarrow 0$, to

168 $$\begin{aligned} \Sigma _{k,\beta }(|\partial \Omega |+A_d\beta |\Omega |)\leqq C(d)|\Omega |^{\frac{d-2}{d}}k^{2/d}. \end{aligned}$$

Taking the limit $\beta \rightarrow \infty$ leads to

169 $$\begin{aligned} \mu _k|\Omega |\leqq C(d)|\Omega |^{\frac{d-2}{d}}k^{2/d}. \end{aligned}$$

This is equivalent to

170 $$\begin{aligned} \mu _k|\Omega |^{2/d}\leqq C(d)k^{2/d}. \end{aligned}$$

$$\square $$

Finally, all is left to do is to prove the previous theorem has the following corollary in dimension $d = 2$. It allows one to transform universal bounds for Steklov eigenvalues into universal bounds for Neumann eigenvalues.

We write $\Omega ^\varepsilon _\beta$ for a domain $\Omega ^\varepsilon$ as constructed earlier whose holes are exactly of radius $r_\varepsilon ^{d-1} = \beta \varepsilon ^{d}$.

### Proof of Theorem 9

We have from Theorem 2 that

171 $$\begin{aligned} \begin{aligned} \lim _{\varepsilon \rightarrow 0} \sigma _k\left( \Omega _\beta ^\varepsilon \right) \left|\partial \Omega _\beta ^\varepsilon \right|&= \Sigma _{k,\beta }(\Omega )\left( \left|\partial \Omega \right| + A_d \beta \left|\Omega \right|\right) \\&= \frac{\left|\partial \Omega \right|}{\beta }{\widetilde{\Sigma }}_{k,\beta } + A_d \left|\Omega \right|{\widetilde{\Sigma }}_{k,\beta }. \end{aligned} \end{aligned}$$

Now, the first term clearly goes to 0 as $\beta \rightarrow \infty$, while, by Theorem 5, we have that

172 $$\begin{aligned} \lim _{\beta \rightarrow \infty } A_d \left|\Omega \right|{\widetilde{\Sigma }}_{k,\beta } = \mu _k(\Omega ) \left|\Omega \right|. \end{aligned}$$

$$\square $$

## Footnotes

1 In the time since the first apparition of this paper as a preprint, this conjecture has been proved false by the first and third-named authors, along with Mikhail Karpukhin [21]. The precise value is in fact ${\widehat{\sigma }}_k^* = 8 \pi k$.
